# Quantifying the relationship between food sharing practices and socio-ecological variables in small-scale societies: A cross-cultural multi-methodological approach

**DOI:** 10.1371/journal.pone.0216302

**Published:** 2019-05-29

**Authors:** Virginia Ahedo, Jorge Caro, Eugenio Bortolini, Débora Zurro, Marco Madella, José Manuel Galán

**Affiliations:** 1 Área de Organización de Empresas, Departamento de Ingeniería Civil, Escuela Politécnica Superior, Universidad de Burgos, Burgos, Spain; 2 CaSEs—Culture and Socio-Ecological Systems research group, Departamento de Arqueología y Antropología, Institución Milá y Fontanals–Consejo Superior de Investigaciones Científicas (CSIC), Barcelona, Spain & Departamento de Humanidades, Universidad Pompeu Fabra (UPF), Barcelona, Spain; 3 Department of Cultural Heritage, University of Bologna, Ravenna, Italy; 4 ICREA, Barcelona, Spain; 5 School of Geography, Archaeology and Environmental Studies, University of the Witwatersrand, Johannesburg, South Africa; 6 INSISOC, Área de Organización de Empresas, Departamento de Ingeniería Civil, Escuela Politécnica Superior, Universidad de Burgos, Burgos, Spain; Universidade Federal de Pernambuco, BRAZIL

## Abstract

This article presents a cross-cultural study of the relationship among the subsistence strategies, the environmental setting and the food sharing practices of 22 modern small-scale societies located in America (n = 18) and Siberia (n = 4). Ecological, geographical and economic variables of these societies were extracted from specialized literature and the publicly available D-PLACE database. The approach proposed comprises a variety of quantitative methods, ranging from exploratory techniques aimed at capturing relationships of any type between variables, to network theory and supervised-learning predictive modelling. Results provided by all techniques consistently show that the differences observed in food sharing practices across the sampled populations cannot be explained just by the differential distribution of ecological, geographical and economic variables. Food sharing has to be interpreted as a more complex cultural phenomenon, whose variation over time and space cannot be ascribed only to local adaptation.

## Introduction

The relationship between species and their environment constitutes a recurring subject matter. Research disciplines such as Behavioural Ecology (BE) and Human Behavioural Ecology (HBE) emerged to provide scientific insights into this issue.

BE investigates how behaviour evolves in relation to ecological conditions, considering these as both the physical and social aspects of the environment [1]. BE has two main lines of investigation: (i) the analysis of how measurable variation in ecological conditions predicts variability in individual behavioural strategies; and (ii) the evaluation of the fitness displayed by individuals as a consequence of their behavioural strategies, (fitness being measured through proxies such as mating success, energetic return, survival rate, or viability). This second approach led to the formation of the so-called “adaptationist stance”. The basic tenet of this line of research is that organisms living in the natural world tend to adjust their behaviour towards an optimum to maximise their fitness under given ecological conditions. This arises from a strict interpretation of the role of environmental pressure within Darwinian natural selection, i.e., that selection, other things being equal, favours genes of individuals who are prone to behave optimally in the specific environmental conditions in which they live [2]. In this context, selection should favour different individual and/or social adaptive mechanisms allowing the bearers to acquire or develop locally adaptive behavioural strategies within a range of environments [3]. Examples of such an evolutionary approach can be found in the works on the development of models to explain foraging (Optimal Foraging Theory) [4–6] or reproductive and demographic behaviour [7].

HBE is defined as the study of human behaviour from the perspective of its adaptiveness, i.e., as the extension and application of the models developed by BE to the particular case of Anatomically Modern Humans [8]. HBE maintains that the study of human behaviour does not entail different explanatory approaches from those used for any other animal species [9]. HBE considers human behaviour as embedded within a given ecological context [8], centring its investigation on production, distribution and reproduction. The first HBE papers appeared in the 1970s and focused mainly on explaining foraging patterns in hunter-gatherer populations [10]. The focus on foragers was mostly due to the long-term perspective offered by such subsistence strategy, and because many researchers considered that small-scale foragers facilitated a straightforward application of Optimal Foraging Theory. In addition, this framework regarded small-scale societies (hereinafter SSSs), and in particular hunter-gatherers, as low-developed groups in which ecological laws could be more easily identified [11,12]. HBE has therefore extended the adaptationist BE paradigm to the study of the relationships and interactions between human populations and their environments [13–15]. Nevertheless, substantial debates have arisen on whether and how far adaptationist approaches are applicable to humans [16], and what elements drive human cultural variability [17]. Its main detractors refer to human adaptationism as over-simplistic and systematically overlooking the role played by human interactions and cultural transmission mechanisms [18,19].

Over the last few decades, there have been several studies aimed at the analysis of human variability in different environmental contexts [20]. More specifically, different comparative studies theoretically grounded in HBE have shown the existence of a relationship between social and ecological parameters, so that human inter-population diversity also reflects adaptation to local habitats [9,21]. However, most HBE studies generally focus on the analysis of a specific phenomenon or a specific social attribute of a single group (foraging, distribution -cooperation and social structure-, mate choice, mating systems, reproductive decisions, parental investment, etc). The foregoing is mainly due to the considerable contribution of Anthropology to HBE, with studies generally representing the field observations of a single field researcher from a single population, usually a single site [8].

One of the topics widely explored from the HBE perspective is that of food sharing, a universal phenomenon that can be found cross-culturally in humans, but which has also been documented in other species such as primates, where it is referred to as an “unresisted transfer of food” among unrelated adults [22]. Studies pointing at the influence of environmental and socio-ecological variables (e.g. availability, distribution and predictability of resources) on food sharing practices are easily found in the literature [21,23].

### Sharing practices background

Food sharing has traditionally been considered a characteristic feature of both human and several non-human societies, and its importance has been highlighted in studies about the evolution of cooperation and sociality, the social division of labour, the development of morality, the transition from earlier hominids to modern humans, and from hunting and gathering to agriculture [24]. In humans, sharing of resources and information is also considered a key aspect in reducing intraspecific competition and increasing population carrying capacity, per-capita growth rate and social stability [25]. Therefore, analysing the cultural variability of this phenomenon and the role played by the different variables involved is essential for understanding human societies.

Sharing happens to be a deeply rooted and complex phenomenon, that ethnographic sources consistently describe as sequences of dynamic events (stages) resulting from highly differentiated forms of individual and group-based interactions [26,27]; interestingly, those stages are combined differently within each society [28,29] constituting an identity trait [30–33]. (Note that by stage we mean the differentiated and successive temporal, spatial and relational steps in which the activities related to sharing practices occur).

These pro-social interactions do not only influence the welfare of the group, but are also encouraged as social and ethical obligations [29] that lead to the development of diverse institutions, mainly of normative kind [34–36], upon which depends the maintenance of social networks [37].

Resource and information sharing have been identified as a long-term strategy to manage risks related with the heterogeneous spatial and temporal distribution of existing resources, as well as to face the imbalances produced between resource availability and population size [25,38]. In addition, factors such as economic crises and colonisation processes are known to exert notable influence on the reinforcement and intensification of sharing behaviours [28,36,39].

Most traditional studies on food sharing among hunter-gatherers focus on the individual characteristics of a specific society (micro-scale analysis) [40–43]. Typically, those studies elaborate on the reasons behind its emergence and development, which are generally associated to resource abundance or to resource pressure [38,44–48].

With the advance of Evolutionary Biology and Ecology, sharing practices were described in terms of fitness and analysed based on their actual or perceived benefits to group physical and social survival [22,23,49,50]. Following this line of research, several models were developed to explain the origin and motivations of sharing [22,49] and their possible link to environmental features [51]. Particularly noteworthy is the work of Winterhalder [52], who, through a modelling approach based on Evolutionary Ecology, showed that major gains in risk reduction by food sharing are achieved in relatively small hunter-gatherer groups, and that the circumstances in which this is possible can be precisely specified in ecological terms. Other studies have pointed to similar dynamics in other species, where food sharing has been observed to occur more commonly when food availability increases [53].

Nevertheless, the progression of research on human food sharing practices has been hampered by the absence of a generalised systematic classification of them, as, despite relevant attempts to establish a typology of resource transfer practices within human societies [29,49,54–58], the development of a systematic description of sharing practices in which every basic unit appears as a mutually exclusive category, with no ambiguity in the terms used, in which any type of transaction can be integrated, and applicable without significant distortions to any human socioeconomic formation, has remained incomplete [26,59,60] until Caro’s doctoral thesis [27]. This fact has restricted research in the field to the predominant traditional and evolutionary approaches [26,59,61,62].

Consequently, in overall terms, all previous research on sharing can be classified into two main categories: (i) single-case analyses with documentary nature and (ii) evolutionary modelling approaches. Remarkably, none of the works falling under these two categories deals with the systematic classification of human food sharing practices, and the vast majority of them neither implement sophisticated data analysis techniques nor perform cross-cultural comparisons to look for generalities.

### Research proposal

In the last few years, there has been a growing interest in cross-cultural studies, mainly due to the creation and development of various global databases presenting cultural, linguistic and environmental data in a unified, standardised and accessible way. Initiatives such as the Human Relation Area Files (eHRAF - http://hraf.yale.edu/), D-PLACE (Database of Places, Language, Culture and Environment - https://d-place.org/ [63]) and Seshat (Global History Databank - http://seshatdatabank.info/ [64]), among others, are becoming key for the future of research on human, social and economic development, and promise to fill the various gaps that still make cross-cultural comparison somewhat difficult.

Accordingly, several inspiring examples of cross-cultural studies can be found in the literature, being the range of phenomena covered significantly varied: Garfield et al. [65] focused on hunter-gatherers and report on the cross-cultural occurrence of different modes and processes of social learning in distinct cultural domains from the ethnographic record; in the work of Sorokowska et al. [66], the authors focused on basic taste preferences in three populations (Polish, Tsimane’ and Hadza), covering a broad difference in diet due to environmental and cultural conditions, dietary habits, food acquirement and market availability; finally, the research by Reyes-García et al. [67] is also an insightful cross-cultural analysis of three subsistence-oriented societies: the Tsimane’ (Amazon), the Baka (Congo Basin) and the Punan (Borneo); in it, they found that variations in individual levels of local environmental knowledge (both culturally transmitted and individually appropriated) relate to individual hunting returns and self-reported health but not to nutritional status, a paradox that is explained through the prevalence of sharing (individuals achieving higher returns to their knowledge transfer them to the rest of the population, and therefore no association between knowledge and nutritional status is found).

Inspired by the increasing number of cross-cultural studies and by the existence of numerous ethnographic examples pointing to the emergence of food sharing practices as a consequence of socio-environmental conditions, such as -among others- the cases of the Yámana hunter-fisher-gatherer society of Tierra del Fuego (Argentina-Chile), the Blackfoot (North-western USA–South-western Canada) and the Copper Inuit (Northern Canada), we decided to conduct a cross-cultural analysis on the possible effect of socio-ecological variables in the emergence of food sharing practices.

For a better understanding of the ethnographic examples that inspired this work, let us elaborate on the Yámana, the Blackfoot and the Copper Inuit cases. The Yámana society was organised in small social units based on households that showed periodical episodes of aggregation. These aggregation episodes occurred in relation to sporadic and unusually high amounts of food resources, such as the stranding of a whale or a high agglomeration of small fish. According to ethnographic sources, the Yámana displayed cooperative behaviours supported by an indirect reciprocity mechanism: whenever an exceptional food resource was discovered, this presence was signalled (through smoke signals) to other groups, bringing together people from wide expanses, so that they could share the food and exchange different types of social capital [38,68,69]. This ethnographically documented example shows how the environmental distribution of resources (the perchance presence of an extraordinary and unpredictable amount of food) can generate specific distribution practices, which are different from those developed during daily life. In addition, it highlights how the temporal variability of food resources can influence the development of specific socioeconomic practices.

In contrast to the Yámana example, the sharing practices of the Blackfoot and the Copper Inuit (both members of the sample of societies explored in this work) were influenced by resource pressure instead of resource abundance. According to [45], periods of reduced food consumption due to lack of game were common among the Blackfoot and other populations of North America, which entailed changes in consumption patterns and a tendency to share equally the limited food returns. With regard to the Copper Inuit, in [46] Damas describes the development of a partnership system as a kind of insurance against food shortages.

Hence, in the light of all the above, we decided to conduct the present study, whose aim is to formally assess to which extent the distribution of traditional food sharing practices observed across the 22 SSSs selected can be explained by: (i) local adaptation to different environmental settings; (ii) the different set of subsistence activities developed by each society in their environmental setting; and (iii) the geographic distance between sampled populations. These possible explanations are not mutually exclusive and, more importantly, there might be other variables affecting/explaining sharing practices, (such as those related to the cultural component [23]). However, the scope of the present work is restricted to the three aforementioned aspects.

A strong relationship between food sharing practices and the environment/subsistence activities may suggest that, to a large extent, they are the result of local adaptations to contextual conditions. If instead geographic distance drives the observed variability in sharing practices, results may suggest a mechanism of adoption of ideas from neighbouring groups, in which similarity in sharing or other cultural practices is dependent on the probability of interaction between communities [70]. If none of the proposed explanations is supported by the empirical distribution observed in food sharing practices, results will make it possible to envisage other processes such as cultural inheritance, or the possibility of functional convergence (i.e., that the different societies develop their sharing practices independently and on grounds of functionality).

The link between socio-cultural traits and environmental settings can be tackled in two ways: (i) through the analysis of purely environmental variables or (ii) by analysing environmental conditions in terms of their social utility (space and temporal resource availability, carrying capacity of the environment, etc.) [71]. We consider the latter to be the most suitable approach for our study, since the different systems of resource redistribution among humans connect the social domain of production with the individual domain of consumption. In this perspective, food-sharing practices have a prominent role in determining how fundamental resources are distributed in SSSs.

With respect to the state-of-the-art analysis conducted in the section entitled “Sharing practices background”, it is important to note that our contribution presents three main differential aspects: (1) it employs Caro’s systematic description of sharing practices [27], which enables to compare the food sharing sequences of different human societies, and to compute quantitative measures to assess the possible relationships between groups in terms of their mutual overlap in sharing practices; (2) it studies human food sharing behaviour from a cross-cultural perspective instead of a local one, (we look for broad patterns at continental scale through the analysis of 22 modern SSSs documented in the Americas and Eastern Siberia); (3) it implements last generation quantitative analysis techniques (exploratory statistics, networks and supervised learning predictive algorithms) to evaluate the role of environmental settings and resource availability in shaping food sharing practices.

## Materials

### Databases and data sources

The focus of this exploratory analysis is on the Americas, a geographically self-contained area with considerable socio-ecological diversity. At the same time, the inclusion of Eastern Siberia provides a useful case-control on the role of local adaptation to Arctic areas, as well as a potential source of information on the peopling of the Americas [72–75].

**Fig 1:**
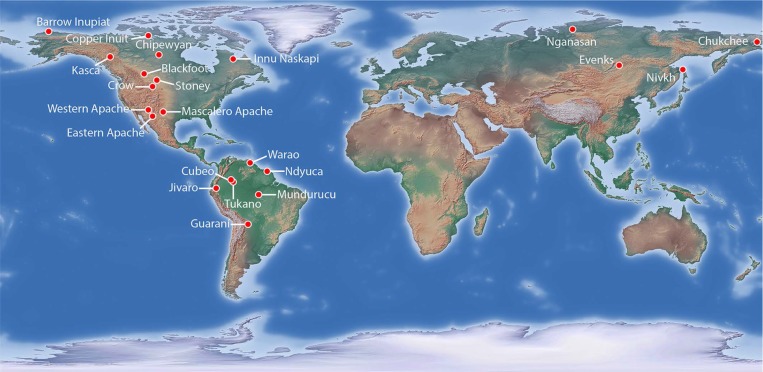
Geographical distribution of the 22 SSSs selected. (Made with Natural Earth). Using ethnographic information extracted from the Human Relation Area Files database (eHRAF - http://hraf.yale.edu/) and other relevant literature (see [27]), we constructed an inventory of the food sharing practices recorded in the 22 SSSs selected. Information related to the environmental and economic variables was extracted from Murdock’s Ethnographic Atlas [76] and/or Binford Hunter-Gatherer [77], available at the Database of Places, Language, Culture and Environment (D-PLACE– https://d-place.org/ [63]). The relation of field and coverage dates for the different variables in each SSS can be found in Table 1; (note that according to eHRAF user guide [78], field date is the date the researcher conducted the fieldwork or archival research that produced the document, and the coverage date is the date or dates that the information in the documents pertains to -often not the same as the field date-).

Having chosen the geographical area, the 22 modern SSSs studied (Fig 1) were selected on the basis of the availability of environmental data, general economic data, and specific information on sharing practices. A database gathering all this information was created (see below the detailed description of all the variables in the database).

**Table 1:** Field and coverage dates of each SSS according to the different data sources.

		Sharing practices information			Environmental and economic variables
Society (eHRAF & literature name)	Society (D-PLACE name)	References	eHRAF Field Date	eHRAF Coverage Date	D-Place Data Field Date
Copper Inuit	Copper Inuit	Damas 1972 [79], 1996 [46]	1962–1963	Pre-contact - late 1960s	1920 [80]
Blackfoot	Blackfoot	Ewers 1955 [45], Nugent 1993 [81]	1941–1951	ca. 1750–1952	1850 [82]
Chipewyan	Dene	Sharp 1981 [83], 1994 [84], VanStone 1963 [85]	1960–1975	1715–1985	1880 [86]
Crow	Crow	Morgan 1959 [87], Frey 2014 [88]	1859	1859	1870 [89]
Guaraní	Guaraní	Reed 1995 [90], Reed & Beierle 1998 [91]	1981–1984	1900-1980s	1900 [92]
Innu Naskapi	Naskapi	Henriksen 1973 [93], Reid 2009 [94]	1966–1968	1900–1973	1890 [95]
Kaska	Kaska	Honigmann & Bennett 1949 [96], Honigmann & Abate 2012 [97]	1943–1945	1940–1945	1920 [98]
Mescalero Apache	Mescalero	Basehart 1970 [99], Basehart & Comm. 1974 [100]	1957–1964	1800–1890	1870 [101]
Mundurucú	Munduruku	Murphy 1960 [102], Murphy & Murphy 1985 [103]	1952–1953	1952–1953	1950 [104]
Stoney	Assiniboine	Snow 1977 [105], Beierle 2002 [106]	1969–1972	1876–1977	1870 [107]
Warao	Warao	Heinen 1973 [108], Heinen & Ruddle 1974 [109], Heinen & Beierle 2001 [110]	1966–1973	1966–1971	1950 [111]
Tukano Makuna	Tucano	Arhem 1981 [112], Beierle 1998 [113]	1971–1973	1971–1973	1950 [114]
Eastern Apache	Chiricaua	Opler 1941 [115], Beierle 2012 [116]	1931–1937	1840–1886	1880 [117]
Jivaro	Shuar	Karsten 1935 [118], Harner 1984 [119], Beierle 2006 [120]	1916–1929	1916–1929	1930 [121]
Western Apache	Western Apache	Perry 1993 [122], Greenfield & Beierle 2002 [123]	no date	nineteenth century - 1980s	1870 [124]
Ndyuka	Ndyuka	Lenoir 1997 [125], Van Wetering & Thoden van Velzen 1999 [126]	1970–1972	1970–1972	1960 [127]
Cubeo Tukano	Cubeo	Goldman 1963 [128]	1939–1940	1939–1940	1940 [129]
Barrow Inupiat	Inupiat	Bodenhorn 2000 [28]	1984–2000	1970–2000	1880 [130]
Nivkh	Nivkh	Shternberg et al. 1993 [131], Austerlitz 2010 [132]	1890–1910	1890–1930	1920 [133]
Nganasan	Nganasan	Ziker 2002 [134], 2007 [135], 2014 [36], Adem 2012 [136]	1992–2014	1992–2014	1930 [137]
Chukchee	Chukchi	Zhornitskaya & Wanner 1996 [138], Ikeya 2013 [139], Krupnik 1987 [140]	2003–2006	Late 19th century-2006	1900 [141]
Evenks	Evenk	Anderson 1991 [142], David et al 2010 [143]		Mid-20th century (1950)-2000	1890 [144]

Regarding Table 1, as might be expected, the field and coverage dates for the different variables across the different SSSs considered are not always coincident. This is mainly due to the intrinsic nature of Anthropology, which renders impossible the concurrent study of all societies in the globe; therefore, cross-cultural databases gather information retrieved by different authors in different field work campaigns, which generally translates into unavoidable time lags.

The 22 SSSs selected and their environments were all documented with coverage dates ranging from ca. 1750 up to 2014. Hence, one may presume the existence of a potential bias due to the differences in the periods of time when data were collected. Nevertheless, it is important to highlight that sharing is characterised by its continuity and stability over time [36], which might be explained because of its key role in the preservation of social networks [37,145], a role that is enacted through the development of various social institutions -mainly normative- [34,36,146] that may include different types of sanctions [35]. By reinforcing these social institutions, traditional sharing is consolidated as an identity trait and solidarity symbol, becoming one of the main cultural features of SSSs [30–33], and thus ensuring its continuity. In addition, traditional sharing of resources and information is also bolstered by other important factors such as resource pressure and/or the processes of western colonisation [25,38]. Many examples in the ethnographic literature show that while these factors result in deep alterations in the field of production, traditional sharing practices are maintained and even intensified in some cases [28,31,36,45–48,147]. Particularly illustrative are the cases of the Huaorani [148] and the Nunavimmiut (Nunavik Inuit) [149]; within the Huaorani community, sharing is maintained -even if they have access to a market- since it meets needs not met through market participation; similarly, the Nunavik Inuit determine an acceptable level of compensation for the exploitation of their region’s minerals, as well as how this compensation will be allocated fairly among their communities, based on their tradition of sharing.

In view of all the above, it can be concluded that although traditional sharing practices may have experienced some minor changes throughout history, they tend to be maintained within SSSs, with less transformations than other cultural, social or economic traits [39]. This fact renders them particularly suitable for cross-cultural studies, as the potential biases related to time lags are well overcome through their stability and continuity over time.

### Information on sharing practices

Food sharing practices in SSSs consist of a sequence of distribution events that start from the moment a resource is obtained. The order in which the different distribution events occur, however, is a distinctive trait, and varies from one society to another, making it difficult to have a cross-cultural comparison when the sequence is considered as a whole, and/or when the order of the events is taken into account.

Therefore, following Caro’s systematic description of food sharing practices [27], we decided to split the sharing sequence into basic stand-alone units (practices) that cannot be further broken into lower-level elements. The result is a set of 14 different basic sharing practices and their systematic description (Table 2; for more detailed information see [27]).

**Table 2:** List of the 14 basic food-sharing practices.

	Code	Practice	Explanation
1	MM	Mutualism	Earn equal shares through cooperative acquisition
2	TT	Tolerated Theft	Communal and free access to the food
3	CC	Communal Consumption	Communal consumption through the celebration of feasts, public events, etc.
4	WD	Women as Distributors	Women are in charge of distributing food
5	OD	Other Distributors	A specific individual such as the chief, shaman or an elder person distributes food
6	RM	Ranked Mutualism	Earn differentiated shares through cooperative acquisition
7	KS	Kin Selection	Give food to close family or distribution within the own household
8	GS	Group Selection	Portions given to every single household of the group
9	NS	Network Selection	Portions given to partners or extended family
10	PR	Prestige	Distribution based on gaining prestige
11	SD	Status Distribution	Food transfers to specific prestigious individuals
12	DS	Demand Sharing	Distribution based on demand
13	RA	Reciprocal Altruism	Asymmetrical distribution based on contingency
14	NN	Necessity	Allocate portions to the neediest

The next step was to identify the basic sharing practices present in the sharing sequence of each of the SSSs selected. (See Table 3. For more detailed information please refer to [27]).

**Table 3:** Basic sharing practices found in the sharing sequence of each society.

Society (eHRAF & literature name)	Society (D-PLACE name)	Sharing practices	Sources
Copper Inuit	Copper Inuit	KS, NS, RA, CC	Damas 1972 [79], 1996 [46]
Blackfoot	Blackfoot	GS, OD, NN, DS	Ewers 1955 [45], Nugent 1993 [81]
Chipewyan	Dene	GS, WD, NS, PR, RA	Sharp 1981 [83], 1994 [84], Van Stone 1963 [85]
Crow	Crow	RM, KS, WD, TT, PR	Morgan 1959 [87], Frey 2014 [88]
Guaraní	Guaraní	KS, RA, NS, SD	Reed 1995 [90], Reed & Beierle 1998 [91]
Innu Naskapi	Naskapi	MM, NN, DS, CC, PR	Henriksen 1973 [93], Reid 2009 [94]
Kaska	Kaska	KS, WD, TT, CC	Honigmann & Bennett 1949 [96], Honigmann & Abate 2012 [97]
Mescalero Apache	Mescalero	RM, GS, PR	Basehart 1970 [99], Basehart & Comm. 1974 [100]
Mundurucú	Munduruku	NS, WD, GS, RA	Murphy 1960 [102], Murphy & Murphy 1985 [103]
Stoney	Assiniboine	KS, GS	Snow 1977 [105], Beierle 2002 [106]
Warao	Warao	KS, SD, NS, CC, RA	Heinen 1973 [108], Heinen & Ruddle 1974 [109], Heinen & Beierle 2001 [110]
Tukano Makuna	Tucano	KS, NS, RA, GS, WD	Arhem 1981 [112], Beierle 1998 [113]
Eastern Apache	Chiricaua	TT, KS, NN	Opler 1941 [115], Beierle 2012 [116]
Jivaro	Shuar	SD, OD, CC, PR	Karsten 1935 [118], Beierle 2006 [120]
Western Apache	Western Apache	MM, DS, NS, PR	Perry 1993 [122], Greenfield & Beierle 2002 [123]
Ndyuka	Ndyuka	KS, WD, NS	Lenoir 1997 [125], Van Wetering & Thoden van Velzen 1999 [126]
Cubeo Tukano	Cubeo	KS, WD, CC, NN	Goldman 1963 [128]
Barrow Inupiat	Inupiat	RM, WD, TT, NN, CC	Bodenhorn 2000 [28]
Nivkh	Nivkh	MM, NN, TT	Shternberg et al. 1933 [131], Austerlitz 2010 [132]
Nganasan	Nganasan	RM, KS, WD, NS, PR, RA, DS	Ziker 2002 [134], 2007 [135], 2014 [36], Adem 2012 [136]
Chukchee	Chukchi	RM, OD, GS, SD, NN, KS	Zhornitskaya & Wanner 1996 [138], Ikeya 2013 [139], Krupnik 1987 [140]
Evenks	Evenk	GS, OD, NN	Anderson 1991 [142], David et al. 2010 [143]

### Environmental variables

The environmental variables considered are climatic and/or ecological proxies that reflect differences between the ecological settings inhabited by the selected SSSs. They include: annual mean temperature (°C), annual temperature variance (°C), temperature constancy, temperature contingency, annual mean precipitation (mm), annual precipitation variance, precipitation constancy, precipitation contingency, distance to the coast (km), elevation (masl) and slope (degrees).

Environmental phenomena expressed by the above climatic/ecological proxies can range from predictable to unpredictable. A phenomenon is completely predictable when it is consistently repeated every year, while it is unpredictable when all states are equally likely in all seasons (see S1 Supporting Information in [63] and [150]). Predictability has two separable components: constancy and contingency. Maximum predictability can be attained as a consequence of either complete constancy, complete contingency or a combination of the two, with respect to time. Complete constancy implies that the phenomenon is the same for all seasons in all years, whereas complete contingency means that the state of the feature is different for each season, but the pattern is the same for all years. Based on this, instead of considering these three variables for our analyses, we worked with constancy and contingency, as predictability can be obtained from the two.

### Economic variables

The selected economic variables are those related to resource richness and resource necessity (in relation to population levels), as well as those related to resource management. More specifically: monthly mean net primary production (measured in grams of carbon uptake per square meter of land per month (gC m-2 month-1)), annual net primary production variance, net primary production constancy, net primary production contingency, population size, and the relative percentage of dependence on different subsistence strategies (hunting, gathering, animal husbandry, fishing, and agriculture).

Population size was considered within the economic variables, since in conjunction with net primary production, it is a good indicator of resource pressure.

For further details on the meaning of each one of these variables and their ranges please refer to S1 Appendix.

By way of summary, Table 4 presents the relation of all the variables involved in the present work and the categories to which they belong.

**Table 4:** Summary of all the variables considered in this study.

Basic sharing practices	Environmental variables	Economic variables
• Mutualism • Tolerated Theft • Communal Consumption • Women as Distributors • Other Distributors • Ranked Mutualism • Kin Selection • Group Selection • Network Selection • Prestige • Status Distribution • Demand Sharing • Reciprocal Altruism • Necessity	• Annual mean temperature (C) • Annual temperature variance (C) • Temperature constancy • Temperature contingency • Annual mean precipitation (mm) • Annual precipitation variance • Precipitation constancy • Precipitation contingency • Distance to the coast (km) • Elevation (masl) • Slope (degrees)	• Monthly mean net primary production • Annual net primary production variance • Net primary production constancy • Net primary production contingency • Population size • Relative percentage of dependence on: • Hunting • Gathering • Animal Husbandry • Fishing • Agriculture

Finally, last but not least, pairwise geographic distance was used as a proxy for the effect that interaction between populations may have on the adoption of sharing practices. This proxy is based on the assumption that populations that are closer in space may exhibit a higher degree of similarity of both genetic as well as non-adaptive cultural traits, which could imply that SSSs geographically closer may have more similar sharing practices [151–153].

## Methods: Data analysis

The set of analyses applied to the dataset described above, and the transformations needed to conduct them, can be summarised as follows:

1. **Exploratory analysis** of the possible relationships between each socio-ecological variable (except for geographic distance) and each basic sharing practice, across the selected SSSs. For that purpose, each socio-ecological variable was split into two groups according to the presence (1) / absence (0) -binary codification- of each of the 14 basic sharing practices, across the 22 SSSs selected. Then, the two groups were compared to ascertain if statistically significant differences exist between them. Different unpaired statistical tests were used for the different cases depending on their particularities: *t*-test, Wilcoxon test, Fligner-Policello test and/or Brunner Munzel test, (further details below).
2. **Sharing similarity network** to verify whether geographically closer societies exhibit more similar sharing practices. In this network, the 22 SSSs were set as nodes, being two nodes linked by an edge if they have in common a specific basic sharing practice. The network was represented on a map of the world in accordance with its geographical location.
3. **Formalization of dissimilarity in food sharing practices** through pairwise Hamming distance. In point 1, the analyses were restricted to evaluating the possible impact of each socio-ecological variable on the presence/absence of each basic sharing practice across the 22 SSSs. However, from point 3 on, the aim is to determine whether larger pairwise differences in the values exhibited by each SSS for the different socio-ecological variables, are linked to greater dissimilarity in sharing practices (greater Hamming distance).

Sharing Hamming distances were calculated pairwise from each of the 22 SSSs to all the rest, considering the whole sequence of food sharing practices -codified as a binary vector with 14 positions, one for each basic sharing practice-. The Hamming distances obtained are quantitative values expressing how distant each pair of SSSs is in terms of sharing.

4 **Transformation of the explanatory variables** into pairwise difference variables, to be able to attain the objective described in point 3. For that purpose, we computed the difference between the values exhibited by every pair of societies for each socio-ecological variable. Pairwise geographic distance was computed as great-circle distance, i.e., the shortest distance between two points on the surface of a sphere, measured along the surface of the sphere.
5 **Exploratory analysis** by means of the Maximal Information Coefficient (MIC), Distance Correlation (dCor) and the Heller-Heller-Gorfine (HHG) measure, to check for the existence of relationships of any type between dissimilarity in sharing practices (Hamming distance) and the pairwise differences (between SSSs) of the socio-ecological variables.
6 **Implementation of supervised learning regression algorithms** to try to predict the dissimilarity in food sharing practices (Hamming distance values) taking as regressors the pairwise differences of the socio-ecological variables. The selected algorithms were Support Vector Machines (SVM) with radial kernel and ensemble methods: random forest, boosting and rotation forest.

### 1. Exploratory analysis

It was conducted to try to identify all possible relationships between each one of the socio-ecological variables and the presence/absence of each one of the basic sharing practices, taking one socio-ecological variable and one basic sharing practice at a time. In this first analysis, the socio-ecological variables remained unchanged, i.e., we worked with the original values recorded for each variable in each SSS, (we did not take pairwise differences in values yet).

Based on the above, geographic distance was not evaluated at this stage, since it is the pairwise geographic distance that is relevant for our study, not the distances to a common origin.

Identifying the existence of any relationships between an independent categorical variable (the presence/absence of a basic sharing practice across the 22 SSSs) and an independent continuous variable (the socio-ecological variables), requires a two-sample statistical test such as the independent *t*-test [154] for two samples. In our analysis, different unpaired two-sample statistical tests were used depending on the details of each case. The rationale behind all these tests, however, is quite similar: first, each socio-ecological variable is split into two groups: the one that presents the basic sharing practice under consideration and the group lacking that feature. Then, the most suitable statistic for each case is calculated, to determine if the means of the two groups are significantly different from each other (at a 0.05 significance level). The independent *t*-test is only suitable when normality and homogeneity of variance can be assumed [155]. For the cases violating one or both assumptions, other tests are needed. If it is the normality assumption that is violated, the most commonly used test is the Wilcoxon-Mann-Whitney test, whose power, according to some authors, appears to be asymptotically superior to that of the *t*-test for real high quality data [156]. If both assumptions are violated, especially recommended are the Fligner-Policello test and the Brunner and Munzel test [155], the latter being generally better according to Fagerland MW et al. [157]. Other authors argue that the Wilcoxon-Mann-Whitney test can be also used when both assumptions are violated, since the Fligner-Policello test and the Wilcoxon-Mann-Whitney test have been found to have roughly similar power [158].

In this exploratory phase, a total of 294 tests were run. In order to overcome the multiple testing problem, we implemented different multiple comparison corrections: two conservative approaches (Bonferroni and Šidàk [159]) and a set of more flexible corrections, namely Holm, Hochberg, Hommel, Benjamini & Hochberg, and Benjamini & Yekutieli [160].

### 2. Construction of a cultural similarity network

To visualise the interrelations between societies, we built a network by setting the 22 SSSs as nodes, and where two nodes are linked by an edge if they have in common a basic sharing practice. Then, to get rid of multiple links, the network was transformed into a weighted network, where the weight of each edge represents the number of links (basic sharing practices) that the two societies have in common. The higher the weight, the greater the similarity between the sharing practices of these two societies.

### 3. Formalization of dissimilarity in food sharing practices

Food sharing practices are a complex phenomenon. Each SSS has its characteristic sharing behaviour, which consists of a sequence of distribution events in which the order of appearance of the basic sharing practices is distinctive, constituting a group identity trait [27,33]. (Recall that by stage we understand the differentiated and successive temporal, spatial and relational steps in which the activities associated with each basic sharing practice occur; and by order of appearance, the position in which every basic sharing practice appears within the sequence [27]).

At this point, a shift in focus was needed to consider all the basic sharing practices constituting a sequence together; hence, each SSS was assigned a vector with as many positions as basic sharing practices -14-, where the presence/absence of each one of them was codified in binary terms, regardless of the order of appearance. With this codification, it seemed reasonable to quantify dissimilarity in food sharing practices through Hamming distance, which, for two vectors of equal length, corresponds to the total number of positions exhibiting different values [161].

The details of the binary codification of sharing practices can be found in S1 Table.

### 4. Transformation of the explanatory variables

After the initial exploratory analysis (where all the socio-ecological variables remained unchanged), the subsequent analyses required transformations to have a coherent framework where all variables could be treated equally. The transformations required consisted in:

A) Formalizing the difference in sharing practices between societies in terms of pairwise Hamming distance -see point 3-. (This resulted in 231 values after removing the diagonal and the symmetrical values of the 22x22 dissimilarity matrix).

B) The calculation of pairwise geographic distances (great-circle distance).

C) Calculating pairwise value differences between societies for the rest of socio-ecological variables (again, 231 values were obtained for each variable after removing the diagonal and the symmetrical values). This is a key point, because it implies that except for geographic distance (where the original value was considered), the rest of the explanatory variables were analysed in terms of the difference in their values (i.e., how does the pairwise difference in value of the socio-ecological variables relate to the pairwise dissimilarity in sharing practices?).

### 5. Exploratory analysis by means of MIC, dCor and HHG

Having transformed all variables, three exploratory statistical tools (namely MIC [162], dCor [163,164] and HHG [165]) were used; these tools were developed to identify a wide range of associations between variables, both functional and not. Although the mathematics behind each tool are significantly different, both MIC and dCor measure the intensity of a relationship of any type, while HHG gives the probability that the relationship truly exists by means of four different *p*-values obtained for four different statistics.

MIC is a recent metric designed to capture the strength of a wide range of associations between variables. However, some authors [163,166] suggest that this statistic may have shortcomings with respect to the properties of equitability and generality, as well as in terms of statistical power for samples of limited size. Therefore, we considered the concurrent use of other measures such as distance correlation (dCor) and HHG to overcome these shortcomings. The results of the three metrics complement each other and give a wider insight into the possible relationships between items. MIC and dCor measure the strength of the association between variables and HHG provides four different *p*-values: *pval*.*hhg*.*sc*, *pval*.*hhg*.*sl*, *pval*.*hhg*.*mc and pval*.*hhg*.*ml*, corresponding respectively to: 1) the sum of Pearson chi-squared statistics from the 2x2 contingency tables considered (sum.chisq); 2) the sum of likelihood ratio (*G* statistic) values from the 2x2 tables (sum.lr); 3) the maximum Pearson chi-squared statistic from any of the 2x2 tables (max.chisq), and the maximum *G* statistic from any of the 2x2 tables (max.lr) [165]; (for the sake of simplicity we have used the nomenclature from the HHG package in R (https://CRAN.R-project.org/package=HHG)).

Regarding the calculation of HHG, we selected Euclidean distance for its computation, as it allows for a straightforward interpretation in the present context of application.

As 22 socio-ecological variables were considered (23 with the Hamming distance in sharing practices itself), 23 comparisons were made in this phase. Therefore, it was necessary to implement some multiple comparison correction procedures, namely Bonferroni, Holm and Hochberg [160].

### 6. Implementation of supervised learning regression algorithms

Up to this point, all the environmental and socio-ecological variables have been considered separately (one at a time); hence, we decided to apply non-linear regression algorithms [167,168] to our dataset of transformed variables (pairwise differences), to check if when taken together, these variables present an explanatory power with respect to the sharing Hamming distance that they do not have when taken separately. The idea behind creating a predictive model is that it may exist a complex pattern simultaneously involving several variables that might explain the output.

In the field of data mining, there is a general consensus that ensemble methods are suitable techniques for dealing with the most difficult problems [169]. The main idea behind the ensemble methodology is to aggregate multiple weighted models in order to obtain a combined model that outperforms every single model in it [170]. In addition, the family of algorithms based on Support Vector Machines (SVMs) has proved to give very good results both for regression and classification [167,171,172]. Therefore, we used both approaches.

An accurate predictive model needs to have a good bias-variance trade-off, which refers to the necessity of a middle-ground solution between a very general model that fails to include important details, therefore lacking accuracy (high bias), and an overfitted model which fails to generalize on new data (high variance). Ensembles are good at finding that compromise since each model in them can be somewhat overfitted, taking under consideration the singular details of its particular training data, but this effect is counteracted by averaging the outputs of all models in the ensemble. According to Fernández et al. [167], random forest is the ensemble method most likely to obtain the best results in different scenarios. Nevertheless, the most suitable model for each case study depends directly on the details of the case, as there is no specific model which outperforms all the others in all cases [173]. Consequently, in this work we implemented -together with random forest- other three high-performance algorithms: two ensembles (boosting [174] and rotation forest [175,176]) and SVM with radial kernel.

Eventually, an Analysis of Variance (ANOVA) test was conducted, to compare the accuracy of the four regression algorithms with that of the prediction of the mean (predicting the average value in all cases), and to see if the differences were statistically significant.

## Results

### Exploratory analysis

Different two-sample statistical tests were applied to the raw values of the socio-ecological variables depending on the particularities of each case (*t*-test, Wilcoxon-Mann-Whitney, Fligner-Policello and Brunner and Munzel).

The results of the 294 tests conducted in this phase can be found in S2 Table.

The details of the multiple comparison corrections can be found in Supporting Information S2 Appendix, where first the value obtained with the two conservative approaches is presented (Bonferroni and Šidàk), and then, Table B collects the *p*-values corrected according to more flexible approaches (Holm, Hochberg, Hommel, Benjamini & Hochberg, Benjamini & Yekutieli).

Even though the aim of this analysis was to detect the possible relationships between the presence/absence of each basic sharing practice (codified in binary terms) and each of the environmental and economic variables considered, for a significance level of 0.05, no significant relationships were found except for the percentage of dependence on animal husbandry and status distribution. In this case, the *p*-value obtained with the Brunner and Munzel test is so low that in accordance with all the multiple comparison corrections implemented, the null hypothesis of equality of means between the two groups -no effect of the socio-ecological variable- has to be rejected.

Because a significant relationship between the percentage of dependence on animal husbandry and status distribution is suggested by only one test (Brunner and Munzel) out of the three tests run on this case (Fligner-Policello, Brunner and Munzel and Wilcoxon-Mann-Whitney), it would be misleading to consider this result as a strong evidence of relationship. On the contrary, it suggests that a relationship may exist between these two variables, although further research on the subject would be needed to check if the relationship continues to be significant when more SSSs are considered.

### Cultural similarity network

The output of this approach is a food sharing similarity network. After positioning the nodes (SSSs) according to their geographic location in terms of latitude and longitude, a qualitative assessment of the visualization obtained was performed. The main conclusion drawn is that simple geographic distance appears not to be related to dissimilarity in sharing practices, as societies from South America are more heavily linked (present links with greater weight) with societies in Siberia and North America than with societies that are closer to them in space.

### Exploratory analysis by means of MIC, dCor and HHG

MIC, dCor and HHG are statistical tools conceived to capture a wide range of associations between variables. MIC and dCor measure the intensity of the association while HHG gives four *p*-values on the probability that the relationship truly exists in reality.

Table 5 presents the values of MIC, dCor and HHG *p*-values obtained for the possible relationships between dissimilarity in sharing practices (Hamming distance) and each one of the transformed socio-ecological variables (pairwise differences between their values).

**Table 5:** Values of MIC, dCor and the four *p-*values provided by HHG -without any multiple comparison correction-.

		Food sharing Hamming distance					
		MIC	dCor	pval.hhg.sc	pval.hhg.sl	pval.hhg.mc	pval.hhg.ml
1	Geographic Distances	0.1748	0.0834	0.9650	0.9530	0.8042	0.9700
2	Annual Mean Temperature Difference	0.2285	0.1756	0.2288	0.2707	0.5954	0.8142
3	Annual Temperature Variance Difference	0.2566	0.1374	0.3616	0.3816	0.2178	0.4316
4	Temperature Constancy Difference	0.1939	0.1612	0.0809	0.0639	0.8012	0.6204
5	Temperature Contingency Difference	0.2311	0.1811	0.0889	0.0789	0.0729	0.1528
6	Annual Mean Precipitation Difference	0.2028	0.1679	0.2567	0.2757	0.7073	0.8751
7	Annual Precipitation Variance Difference	0.1930	0.1345	0.2138	0.2248	0.1838	0.3357
8	Precipitation Constancy Difference	0.1752	0.0890	0.5534	0.5724	0.5145	0.5135
9	Precipitation Contingency Difference	0.1924	0.1483	0.2058	0.2018	0.6793	0.4915
10	Distance to Coast Difference	0.1974	0.1138	0.5065	0.5085	0.8022	0.7932
11	Elevation Difference	0.2088	0.1159	0.1079	0.0959	0.1259	0.4955
12	Slope Difference	0.1974	0.1187	0.2138	0.2398	0.4396	0.6783
13	Hunting Difference	0.1356	0.0979	0.4016	0.4046	0.1269	0.1918
14	Gathering Difference	0.1471	0.1009	0.3207	0.3417	0.5754	0.8252
15	Animal Husbandry Difference	0.0622	0.1764	0.4246	0.4496	0.5674	0.5784
16	Fishing Difference	0.1382	0.1160	0.8412	0.8641	0.7942	0.9091
17	Agriculture Difference	0.1352	0.1769	0.0839	0.0659	0.1958	0.0310
18	Monthly Mean Net Primary Production Difference	0.1911	0.1808	0.4176	0.4156	0.9600	0.9191
19	Annual Net Primary Production Variance Difference	0.1790	0.1390	0.2038	0.2068	0.3057	0.4535
20	Net Primary Production Constancy Difference	0.1985	0.1643	0.4406	0.4466	0.6603	0.3357
21	Net Primary Production Contingency Difference	0.1946	0.0885	0.4745	0.4785	0.5564	0.0719
22	Population Size Difference	0.1562	0.1012	0.6723	0.6723	0.2498	0.4745
23	Food sharing Hamming Distance	0.9924	1	0.0010	0.0010	0.0010	0.0010

Since there are four *p*-values (HHG has four different *p*-value calculation procedures), four tables with the corrected the *p*-values according to Bonferroni, Holm and Hochberg multiple comparison corrections were obtained -one table for each- (see Supporting Information, Tables C, D, E and F in S3 Appendix).

At a 0.05 level of significance, no significant relationships were found between dissimilarity in sharing practices and any of the pairwise differences in value of the socio-economic variables considered. Thereupon, the hypothesis that the more different the socio-ecological variables of two SSSs, the more dissimilar their sharing practices, is not underpinned by the statistical evidence provided by MIC, dCor and HHG.

### Implementation of supervised learning regression algorithms

At this point, we implemented 4 different high-performance regression algorithms: random forest, boosting, rotation forest and SVM with radial kernel. Then, with an ANOVA test, we compared the results of each of them to the prediction of the mean (predicting the average value in all cases).

The results analysed with the ANOVA test were obtained through ten-fold nested cross-validation [177,178] (further details on the results obtained for each fold in S3 Table). The ANOVA test (see results in Table 6) showed that the null hypothesis of equal means across the five algorithms cannot be rejected for a common level of significance (0.05). This means that no pattern relating the output with the explanatory variables was detected, as top prediction algorithms trained on the data were indeed unable to reach higher accuracy than that of the prediction of the mean. Therefore, our proposition that all the regressors (the socio-ecological variables in pairwise difference terms) taken together, could have an explanatory power with respect to the sharing distance that they do not have separately, is not supported by empirical evidence; for a common level of significance (0.05), we cannot reject that the differences may be due to randomness.

**Table 6:** ANOVA table.

	Df	Sum Sq	Mean Sq	F value	Pr(>F)
**Model**	4	1.82	0.4557	0.37	0.829
**Residuals**	45	55.46	1.2325		

ANOVA test conducted on the MSE obtained by means of 10-fold nested-cross-validation for random forest, boosting, rotation forest, SVM with radial kernel and the prediction of the mean. The null hypothesis of equality of means across all of them cannot be rejected for alpha = 0.05.

This result validates our previous findings and confirms the lack of relationships between the observed distribution of sharing practices and the environmental and socio-ecological variables considered.

## Discussion

The results from our analyses point to a generalised lack of statistically significant relationships between food sharing practices and the considered environmental and socio-ecological variables, at the chosen scale of analysis and across all methodologies implemented, i.e.: (1) in terms of direct relationship between each basic sharing practice and each explanatory variable; (2) regarding the network approach, as the sharing similarity network does not support the hypothesis that sharing practices of geographically closer populations may be more similar; (3) in terms of the possible relationships between the pairwise differences in the values of the explanatory variables and the pairwise Hamming distances between sharing practices; and (4) when implementing supervised learning regression algorithms to look for complex patterns simultaneously involving several variables, since no pattern between the regressors (in pairwise difference terms) and the distance in sharing practices was found.

A positive result, however, was obtained with approach (1) for the possible relationship between the percentage of dependence on animal husbandry of a SSS and the presence of sharing practices dominated by status distribution. Since only one test out of three pointed to a significant relationship, this result should be interpreted as a suggestion of relationship, not as a strong evidence of it.

In the literature, several authors pointed to a connection between the beginning of pastoralism (with the important surplus generated by cattle and/or sheep/goats) and the emergence of social stratification linked to status [179]. This result is in accordance with other modelling approaches [180] which suggest that institutionalized social inequality in non-coercive circumstances might arise due to a limited number of asymmetries in a system, such as the control over productive resources or of socially significant information. The outcome of these asymmetries would be the concentration of wealth (or power) in a segment(s) of the social group. In addition, the models in [180] suggest that such asymmetries can be self-reinforcing and therefore, quite stable over time. An ethnographic example illustrating this phenomenon can be found in the Kalahari desert, where access to water-storing melons and domestic animals led to wealth inequality and increased polygyny [181], which is linked to stratification.

Hence, (aside from the suggestion of relationship between animal husbandry and status distribution), based on the results of our analyses we cannot reject the null hypothesis of independence between the selected socio-ecological variables and food sharing practices at the current scale of analysis. There are several reasons that may account for this absence of non-random relationships and which may be grouped under two main categories: (a) missing proxies and (b) additional possibilities.

Regarding missing proxies, it should be emphasized that food sharing practices are a multifaceted phenomenon resulting from the interaction of numerous intertwined mechanisms. The present contribution, because of the HBE approach selected, focused on environmental and socio-ecological variables, leaving aside other factors that may be explicative. However, in the light of the results obtained (no statistically significant relationships found), one may expect that it is the unconsidered proxies that might explain the cross-cultural differences in food sharing practices.

Among the set of possible missing proxies, it could be of interest to consider: (i) the use of a different scale of analysis -particularly a lower one-; (ii) the inclusion of the stages and/or the order in which the basic food sharing practices are performed within the sharing sequence; and (iii) the examination of the processes of cultural transmission and cultural diffusion, as well as the possible mismatch between the context where practice emerged and the context where it is implemented.

### (i) The scale of analysis

The selection of an adequate scale of analysis is critical for the emergence of robust patterns of change in socio-ecological variables, which can then be compared with variability in sharing practices. It is necessary that the scale of analysis coherently articulates with the hypothesis to test, and that it is compatible with the methodology selected. This work was conducted at a macro-scale, i.e., with a sample of SSSs scattered at continental level. Nonetheless, the absence of relationships at this scale of analysis (cross-continental) does not imply that such relationships do not exist at other scales. In fact, Ember et al. showed in [182] that at a worldwide scale, patterns in food sharing can be observed. More precisely, they found that societies subject to more resource stress share more frequently. At lower scales of analysis, such as subcontinental, regional or with a smaller-sized sample of societies, patterns may also be found. A good example of it is the work conducted by Patton [183] between households of Achuar, Quichua and Zapara speakers in Conambo -an indigenous community of horticultural foragers in the Ecuadorian Amazon-. In it, it is stated that transfers of meat in Conambo are best explained by multiple adaptive strategies, many of which are better understood with reference to the political context. Conambo is a game-rich environment (resource richness), allowing for small meat-sharing networks and direct accounting and policing of transfers. As a result, hunters in Conambo exercise control over meat transfers, can more easily practice conditional giving, and target meat transfers to reciprocating households, kin and political allies.

### (ii) The inclusion of the stages constituting the food sharing sequence

Considering the stages in which the whole sharing sequence can be divided is to some extent related to the scale of analysis too. These stages denote the order in which individuals share the obtained resources across the different spheres within the kinship or communal network [27]. Thus, food sharing stages constitute at the same time scales of analysis such as close vs. extended kinship [184].

Although, as discussed earlier, many of the basic sharing practices constituting a sharing sequence are observed cross-culturally, the order in which those basic practices are performed is specific of each group and can be considered an identity trait [27,33]. Therefore, the observation units chosen may have had a limiting or biasing effect on the analyses conducted. We focused on the presence/absence of the 14 basic sharing practices [27] in the whole sharing sequence of each SSS, regardless of the order of appearance. However, it could be argued that the main feature to consider should be the stage at which each practice takes place (what would require comparing stages), or that it is the whole sequence with its intrinsic order that should be considered, (which would imply whole-sequence comparisons).

### (iii) Cultural diffusion, cultural transmission and the mismatch argument

Human cultural variability often depends on non-environmental or non-adaptive mechanisms shaping social behaviour. Hence, for a more comprehensive understanding of cultural phenomena -and specifically of sharing practices-, the competing effects of a great variety of processes of cultural transmission and cumulative cultural change should be considered.

Since the emergence of Dual Inheritance Theory and the study of gene-culture coevolutionary processes [185,186], relevant literature defines cultural transmission as the process by which information is copied, imitated and learnt among conspecifics of the same generation, and passed on to the following generations. Mechanisms underpinning the distribution observed in cultural and behavioural traits may be for example related to the movement of people (i.e. a *demic diffusion*, by which material and immaterial concepts move following the migration of humans carrying them) and, in some cases, to gradual or abrupt population replacement. Alternatively, the spread of ideas and the exchange of information between neighbouring individuals and groups may take place without necessarily entailing migration events or population replacement (i.e. *cultural diffusion*). The above-mentioned scenarios are not mutually exclusive. Both have a relative impact on the total variability recorded in empirical observations that may be formally ascertained [187–191]. It should be stressed that a scenario based on cultural diffusion implies a longer temporal scale in which cultural information is gradually passed on from one group to the next until a cultural or social barrier is encountered. In addition, spatially closer populations interact more often and more intensely than populations located further apart [151–153,192]. The iteration of this process makes geographic distance a good proxy of cultural (or biologic) dissimilarity and generates geographic clines.

Another plausible explanation for the absence of relationships found -which is also related to demic diffusion processes- is the possible mismatch [9] between the context where a cultural trait emerged/developed and the context where it was observed. This may be due not only to population movements, but also to changes in the environment (either for natural or anthropic reasons). Remarkably, mismatch arguments have been claimed to be larger in the case of human societies when compared to other species due to the specific role played by technology [193].

To illustrate all these ideas in the context of sharing, we can think of a society whose sharing practices were developed in a specific environmental setting and which were later exchanged due to proximity to other social groups, or which eventually reached areas far from their geographic origin through migration. In the ethnographic record, both contacts with other groups and/or migratory events are documented for some of the 22 SSSs studied in this work, such as -among others- the Crow, the Blackfoot and the Stoney (all located in the present USA). The Crow are documented to have performed westward migratory processes early in the eighteenth century; as a consequence, they came into continuous contact with other social groups -both for warfare and/or trade-, which possibly resulted in the exchange of different cultural and socioeconomic traits [194,195]. Migration is also recorded among the Blackfoot. According to [196], the Blackfoot tribes were not created in the land which they inhabited at the end of the nineteenth century; probably within 200 years from Grinnell’s publication, the Blackfoot were not plains people, but lived far to the northeast, possibly near or north of Lesser Slave lake. Something similar is reported for the Stoney; in [197] we find that the Stoney may have been in the foothills west of Edmonton by about 1650 and that a mid-17^th^ century entry into the area would roughly coincide with the westward push of Cree and Stoney from around Lake Winnipeg, which presumably began about 1670 and for which two different migration routes into the Rocky Mountains are suggested; later, pressures from adjacent groups may have helped them move further west.

**Fig 2:**
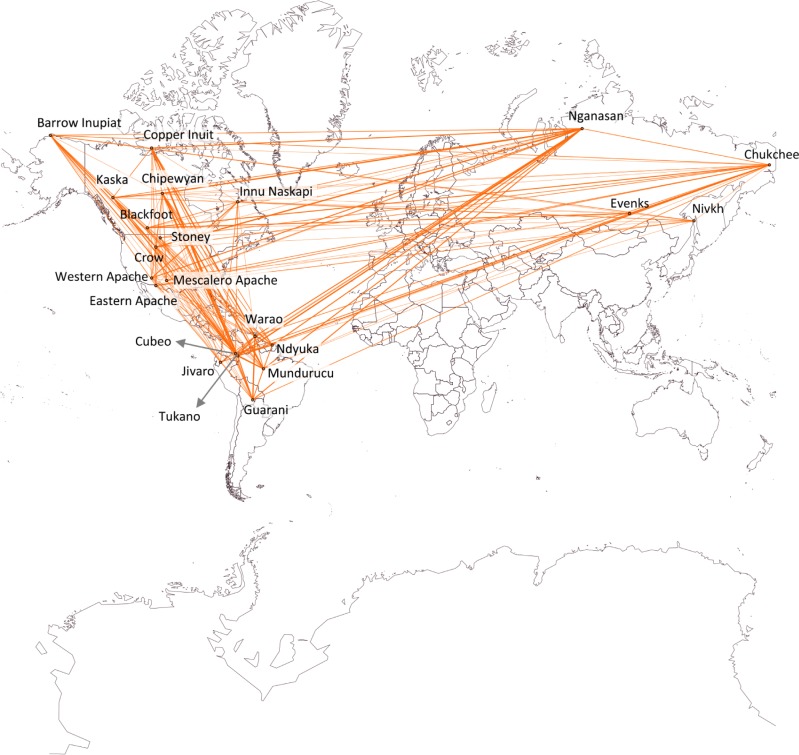
Sharing similarity network. (Made with Gephi GeoLayout and Map of Countries plugins. The maps from Map of Countries plugin were provided by thematicmapping.org).

In view of all the above, the absence of significant relationships between sharing practices and environmental/ecological variables hints at a marginal role played by adaptation to localised conditions and subsistence strategies devised to face different selective pressures. At the same time, the lack of correlation between dissimilarity in sharing practices and pairwise geographic distance suggests that interaction between groups, horizontal exchange of information, and cultural diffusion may not be the key mechanisms underlying the distribution of sharing practices across the study area; a representative example supporting this assertion is that of the Cubeo and the Tukano (both in the present Colombia), whose sharing practices are significantly different despite being extremely close in space (see Fig 1 and Table 3). Migration could then have had a critical role in shaping the observable distribution of food sharing practices, as it is suggested by the strong similarity found between some South American and North-western American/Siberian societies (Fig 2); particularly noteworthy is the example of the Chipewyan (Canada) and the Mundurucú (Brazil), whose sharing practices are almost identical except for the fact that distribution based on prestige (PR) is only performed among the Chipewyan (Table 3). Thereupon, human groups may have developed sharing practices in a specific context and may have moved throughout the study area too quickly for a geographic gradient to form [151,152].

Beyond their emergence in specific socio-ecological conditions, food-sharing practices display a clear component of inherited behavioural dynamics connected to social organization. Thus, a worthwhile future research line would consider the study of common ancestry between pairs of sampled populations, to quantify the relative effect of demic diffusion as opposed to cultural diffusion and mere functional convergence (i.e. independent development of cultural traits or behaviours without inheritance or exchange of information) [198].

Leaving aside the possible role played by missing proxies and unknown variables, the obtained results might be interpreted in the light of two additional arguments: (1) niche construction theory and (2) Galton’s problem.

### (1) Niche construction theory

Niche construction theory (NCT) is a fledgling branch of evolutionary biology that places emphasis on the capacity of organisms to modify natural selection in their environment and thereby act as co-directors of their own, and other species’ evolution [199].

Human niche construction may be uniquely potent, being the capacity for technology and culture a critical factor underlying such potency.

Mathematical models have shown that niche construction due to human cultural processes can be as powerful as niche construction due to biological evolution [200], and, what is more, that because cultural processes typically operate faster than natural selection, cultural niche construction probably has more profound consequences than gene-based niche construction [199]. There is now little doubt that human cultural niche construction has co-directed human evolution [201], and that cultural niche construction can modify the selection of human genes and drive evolutionary events [200,202,203]. Therefore, the relationship with the environment is bidirectional and human activities do not only modify the environment, but also influence biological selection processes as a consequence of their cultural behaviour. There may be instances of local cultural adaptations, which produce a threefold feedback over time on cultural variability, ecological variables, and the genetic pool of those specific populations generating cultural niches [204]. Hence, human cultural niche construction may also have its part in the explanation of the results obtained for sharing practices.

### (2) Galton’s problem

Concerning the power of statistical inference in cross-cultural studies, Galton’s problem (i.e., that cultural variables may or may not be independent from one another) should also be taken into consideration; what Galton’s problem points out is that common ancestry or diffusion may make sample correlations more significant statistically than they would otherwise be, (see [205], Galton's Problem in cross-cultural research). Its main implication is the need to be cautious when drawing inferences from statistical cross-cultural studies, since if variables are not independent, they can give rise to spurious correlations.

With regard to the present case study, as only a positive result was obtained for the relationship between the percentage of dependence on animal husbandry and status distribution, the only precision to be made (as previously pointed) is that our results suggest the existence of such relationship, but do not provide strong evidence of it.

Eventually, we would like to conclude with some brief reflections on cross-cultural studies. There has been much debate around cross-cultural research and the legitimate or illegitimate nature of this type of studies [206]. Four are the main objections argued: the supposed incomparability of cultural traits (cultures are unique and therefore not comparable), the supposed incomparability of units of analysis (societies), the supposed impossibility of unbiased sampling (ethnographic and archaeological studies are inextricably linked to systematic biases related to the very different perspectives adopted by different data collectors over time, as well as in different cultural contexts) and Galton’s problem [205]. The answers to these four objections can also be found in [205], where, in overall terms, what the authors claim is that it is easy to measure variables cross-culturally even if the data (ethnographic, archaeological) are qualitative, and that it is possible to sample the universe of human societies in an unbiased way so that test results can be generalized to all of human experience. In short, their main conclusion in [205,206] is that cross-cultural analysis enables to go beyond case-related particulars and provides results explaining global phenomena that are more generally valid and more easily generalizable than those coming from single-case studies, as no type of research except for cross-cultural studies can say that a result is likely to be true for the world, because only cross-cultural research attaches a worldwide probability to a result.

Now that we are in the era of big data, data analysis and data mining, the software available renders multivariate analysis an easy task. Therefore, the best we can do to achieve a deep understanding of cultural phenomena is to go beyond the particularities of each case and to test our theories cross-culturally. Consequently, even if the use of a continental scale of analysis has previously been argued as a plausible explanation for the lack of relationships found in this work, it is clear that further analyses similar to the present one are needed to consolidate a research line devoted to increasing our global knowledge of social phenomena and to reaching empirically supported, theoretically laden generalizations.

## Conclusions

The overarching aim of this work was to formally explore from a cross-cultural perspective the influence that ecological and economic conditions may have on the development of food sharing practices in human societies. The main results obtained from this study may be summarised as follows:

- At a continental scale focused on the Americas and Siberia, a generalised lack of statistically significant relationships between food sharing practices and the considered socio-ecological variables was found across all methodologies implemented. A single positive result was obtained, which suggested the possible existence of a relationship between the percentage of dependence on animal husbandry and the presence of sharing practices dominated by status distribution.
- The hypothesis that food sharing practices of geographically closer populations may be more similar is not supported either by any of the analyses conducted.

Nevertheless, these results do not exclude the possibility that at a different scale of analysis other relationships may exist, as we know that the chosen scale, the systematic description adopted, and the approach selected, may have had an impact on the strength of the patterns that we were able to identify. Therefore, even if it is out of the scope of the present paper, the use of a different scale of analysis or the inclusion of the stages and/or the order of performance of each basic sharing practice would be worthwhile future research issues.

Furthermore, it would be also strongly recommendable to account for the effects of other sociocultural variables (such as social organization, differences between matrilineality and patrilocality, gender issues, etc.), as well as for the effects of cultural transmission and cultural diffusion processes. Regarding this second aspect, it would be interesting to investigate cultural inheritance and demic migration models through common ancestry between population pairs, to check their relative impact on the observed distribution of food sharing practices.

## Supporting information

S1 Table Binary codification of the presence/absence of the basic sharing practices in the sharing sequences of the 22 SSSs considered. (DOCX)

S2 Table *P*-values for the different independent two-sample statistical tests. The cases where the *t*-test was applied are in white, those where only the Wilcoxon-Mann-Whitney test was applied are in green, and in light pink we can find the three *p-*values obtained for Fligner-Policello (in bold), Brunner and Munzel (in italics) and Wilcoxon-Mann-Whitney (in ordinary font) respectively.
(DOCX)

S3 Table Mean squared error per fold obtained for the different regression models implemented. Mean value over ten folds, standard deviation and standard error in the shadowed rows of the table.
(DOCX)

S4 Table Complete database with all variables considered (raw data). (XLSX)

S1 Appendix Clarification of some concepts related to the socioecological variables selected. (DOCX)

S2 Appendix Multiple comparison corrections. (DOCX)

S3 Appendix HHG corrected *p*-values in ascending order. (DOCX)
