# Supplementary material for: Quantifying the relationship between food sharing practices and socio-ecological variables in small-scale societies: A cross-cultural multi-methodological approach
Source: PLoS One. 2019 May 29;14(5):e0216302. doi: 10.1371/journal.pone.0216302 (PMC6541262; doi:10.1371/journal.pone.0216302)
Supplement: S2 Table — The cases where the t-test was applied are in white, those where only the Wilcoxon-Mann-Whitney test was applied are in green, and in light pink we can find the three p-values obtained for Fligner-Policello (in bold), Brunner and Munzel (in italics) and Wilcoxon-Mann-Whitney (in ordinary font) respectively. (DOCX) [file pone.0216302.s002.docx]

|  | **MM** | **TT** | **CC** | **WD** | **OD** | **RM** | **KS** | **GS** | **NS** | **PR** | **SD** | **DS** | **RA** | **NN** |
| --- | --- | --- | --- | --- | --- | --- | --- | --- | --- | --- | --- | --- | --- | --- |
| Annual mean temperature | 0,586 | 0,401 | 0,734 | 0,744 | 0,468 | 0,058 | 0,550 | 0,954 | 0,262 | 0,447 | 0,305 | 0,262 | 0,680 | 0,142 |
| Annual mean precipitation | 0,718 | 0,649 | 0,945 | **0,260** *0,318* 0,262 | 0,774 | 0,164 | 0,582 | 0,868 | 0,556 | 0,630 | 0,300 | 0,118 | 0,731 | 0,267 |
| Annual precipitation variance | 0,787 | 0,493 | 0,731 | 0,744 | 1,000 | 0,164 | 0,821 | 0,868 | 0,471 | 0,680 | 0,141 | 0,141 | **0,783** *0,827* 0,783 | 0,297 |
| Annual temperature variance | 0,408 | 0,704 | 0,767 | 0,471 | 0,652 | 0,249 | 0,346 | 0,565 | 0,601 | 0,407 | 0,195 | 0,262 | 1,000 | 0,365 |
| Precipitation constancy | 0,549 | 0,807 | 0,114 | 0,043 | 0,817 | 0,636 | 0,336 | 0,619 | 0,385 | 0,758 | 0,116 | 0,594 | 0,957 | 0,434 |
| Precipitation contingency | 0,857 | **0,448** *0,334* 0,446 | 0,407 | 0,794 | 0,967 | 0,058 | 0,821 | 0,570 | 0,324 | 0,307 | 0,262 | 0,118 | 0,447 | 0,570 |
| Temperature contingency | 0,005 | 0,446 | 0,123 | 0,021 | 0,902 | 0,820 | 0,050 | 0,525 | 0,144 | 0,185 | 0,098 | 0,118 | 0,123 | 0,188 |
| Temperature constancy | 0,464 | **0,767** *0,673* 0,762 | 0,490 | 0,471 | 0,538 | **0,251** *0,172* 0,249 | 0,381 | 0,482 | 0,601 | 0,447 | 0,227 | 0,227 | 1,000 | 0,365 |
| Distance to coast | 0,146 | 0,468 | 0,017 | 0,870 | 0,445 | 0,796 | 0,985 | 0,004 | 0,470 | 0,867 | 0,301 | 0,525 | 0,818 | 0,724 |
| Elevation | 0,857 | 0,595 | 0,162 | 0,357 | 0,538 | 0,446 | 0,628 | 0,525 | 0,144 | 0,267 | 0,434 | 0,774 | 0,041 | 0,815 |
| Slope | 0,464 | **0,446** *0,557* 0,446 | 1,000 | 0,948 | 0,227 | 0,493 | 0,821 | 0,764 | 0,126 | 0,407 | 0,967 | 1,000 | 0,026 | 0,868 |
| % dependence on hunting | 0,640 | 0,805 | 0,377 | 0,847 | 0,872 | 0,344 | 0,443 | 0,355 | 0,371 | 0,038 | 0,045 | 0,008 | 0,743 | 0,963 |
| % dependence on gathering | 0,386 | 0,635 | 0,269 | 0,007 | 0,793 | 0,387 | 0,570 | 0,854 | 0,178 | 0,902 | 0,726 | 0,919 | 0,117 | 0,648 |
| % dependence on animal husbandry | 0,968 | 0,077 | 1,000 | 0,177 | **0,135** *0,310* 0,135 | 0,633 | 0,977 | 0,340 | 0,842 | 0,395 | **0,002** *0,00003* 0,002 | 0,210 | 0,880 | 0,964 |
| % dependence on fishing | 0,947 | 0,167 | 0,026 | 0,268 | 0,349 | 0,785 | 0,941 | 0,508 | 0,902 | 0,126 | 0,687 | 0,076 | 0,145 | 0,475 |
| % dependence on agriculture | 0,914 | **0,048** *0,003* 0,048 | 0,899 | 0,934 | 0,881 | 0,101 | 0,936 | 0,933 | 0,289 | 0,635 | 0,092 | 0,332 | 0,959 | 0,222 |
| Annual net primary production variance | 0,271 | 0,844 | 0,563 | 0,420 | 0,504 | 0,072 | 0,579 | 0,674 | 0,759 | 0,553 | 0,676 | 0,384 | 0,627 | 0,837 |
| Monthly mean net primary production | 1 | 0,319 | 1,000 | 0,556 | 0,774 | 0,048 | 1 | 0,764 | 0,235 | 0,783 | 0,141 | 0,342 | 0,783 | 0,145 |
| Net primary production constancy | 0,953 | 0,236 | 0,599 | 0,535 | 0,978 | 0,028 | 0,820 | 0,848 | 0,196 | 0,752 | 0,218 | 0,497 | 0,757 | 0,100 |
| Net primary production contingency | 0,281 | 0,328 | 0,407 | 0,630 | 0,298 | 0,725 | 0,497 | 0,441 | 0,200 | 0,924 | 0,262 | 0,384 | 0,148 | 0,685 |
| Population size | 0,771 | 0,096 | 0,396 | 0,566 | 0,310 | 0,433 | 0,593 | 0,701 | 0,314 | 0,849 | 0,005 | 0,330 | 0,931 | 0,321 |

S2 Table. *P*-values for the different independent two-sample statistical tests.

The cases where the *t*-test was applied are in white, those where only the Wilcoxon-Mann-Whitney test was applied are in green, and in light pink we can find the three *p-*values obtained for Fligner-Policello (in bold), Brunner and Munzel (in italics) and Wilcoxon-Mann-Whitney (in ordinary font) respectively.
