# Supplementary material for: Quantifying the relationship between food sharing practices and socio-ecological variables in small-scale societies: A cross-cultural multi-methodological approach
Source: PLoS One. 2019 May 29;14(5):e0216302. doi: 10.1371/journal.pone.0216302 (PMC6541262; doi:10.1371/journal.pone.0216302)
Supplement: S3 Table — Mean value over ten folds, standard deviation and standard error in the shadowed rows of the table. (DOCX) [file pone.0216302.s003.docx]

|  | **Test MSE** | | | | |
| --- | --- | --- | --- | --- | --- |
| **Fold** | **Random Forest** | **Boosting** | **Rotation Forest** | **SVM radial kernel** | **Prediction Mean** |
| 1 | 3.44149 | 5.07572 | 4.53235 | 4.40609 | 2.62766 |
| 2 | 4.50670 | 4.62127 | 3.03646 | 4.71402 | 3.74307 |
| 3 | 6.96670 | 2.49571 | 3.92135 | 7.46012 | 2.65423 |
| 4 | 3.09195 | 3.25791 | 3.55866 | 3.46720 | 3.25257 |
| 5 | 2.44851 | 3.50723 | 4.09776 | 2.33045 | 4.00657 |
| 6 | 4.93425 | 5.09263 | 4.24854 | 4.82808 | 5.57442 |
| 7 | 2.48567 | 2.56450 | 2.83396 | 2.41279 | 2.59193 |
| 8 | 3.38583 | 3.31633 | 3.99298 | 3.68254 | 4.67249 |
| 9 | 4.37364 | 4.13220 | 4.32482 | 3.83692 | 3.98455 |
| 10 | 4.34482 | 3.00203 | 3.30120 | 4.76361 | 3.87294 |
| **Mean** | 3.99796 | 3.70655 | 3.78481 | 4.19018 | 3.69804 |
| **SD** | 1.35003 | 0.97032 | 0.57490 | 1.46333 | 0.96249 |
| **SE** | 0.42692 | 0.30684 | 0.18180 | 0.46275 | 0.30437 |

S3 Table. Mean squared error per fold obtained for the different regression models implemented.

Mean value over ten folds, standard deviation and standard error in the shadowed rows of the table.
