# Supplementary material for: Quantifying the relationship between food sharing practices and socio-ecological variables in small-scale societies: A cross-cultural multi-methodological approach
Source: PLoS One. 2019 May 29;14(5):e0216302. doi: 10.1371/journal.pone.0216302 (PMC6541262; doi:10.1371/journal.pone.0216302)
Supplement: S1 Appendix — (DOCX) [file pone.0216302.s005.docx]

**S1 Appendix – Clarification of some concepts related to the socioecological variables selected**

**Net Primary Production**

Available resources in a particular environment can be quantified as Net Primary Production (NPP, i.e., the rate at which all the plants in an ecosystem produce net chemical energy after transpiration; [a]) and Net Secondary Production (NSP, i.e., the generation of biomass by heterotrophic organisms in a system). These measures are correlated (at least in the case of herbivores [b]), which makes NPP alone a good proxy for both.

[a] Lancelotti C, Zurro D, Whitehouse NJ, Kramer KL, Madella M, García-Granero JJ, et al. Resilience of small-scale societies’ livelihoods: a framework for studying the transition from food gathering to food production. Ecol Soc. 2016;21: art8. doi:10.5751/ES-08757-210408

[b] Coe MJ, Cumming DH, Phillipson J. Biomass and production of large African herbivores in relation to rainfall and primary production. Oecologia. 1976;22: 341–354. doi:10.1007/BF00345312

**Percentage of dependence on different subsistence economies**

As far as the percentage of dependence on different subsistence strategies is concerned, it is important to note that the data available in D-PLACE were divided into categorical classes, each one of them ranging from a low value to a high value (in percent). As dealing with categorical variables implied a significant increase in noise, a transformation of the percentages was performed to obtain numerical values ranging from 0 to 100.

Transformation of the percentages of dependence on subsistence economies

As highlighted above, the information provided by D-PLACE (Ethnographic Atlas) on the percentages of dependence on subsistence economies was divided into nine groups instead of being a continuous variable. The categories provided can be seen in S1 Table. To ease the analysis of these variables, the following transformations were performed:

1. Instead of the given ranges, the mean value of each range was taken.
2. For each SSS, the mean percentage values assigned to each subsistence economy were added up, which resulted in a total value.
3. A proportion was established by equalling the total value for each society to the maximum value on the desired range, i.e., 100.
4. Eventually, in order to have a scale ranging from 0 to 100, all the percentages of dependence on subsistence economies were transformed by applying the corresponding proportion factor.

|  | **range** | **mean value** |
| --- | --- | --- |
| 1 | 0 to 5 | 2,5 |
| 2 | 6 to 15 | 10,5 |
| 3 | 16 to 25 | 20,5 |
| 4 | 26 to 35 | 30,5 |
| 5 | 36 to 45 | 40,5 |
| 6 | 46 to 55 | 50,5 |
| 7 | 56 to 65 | 60,5 |
| 8 | 66 to 75 | 70,5 |
| 9 | 76 to 85 | 80,5 |

Table A. Categories for the percentage of dependence on subsistence economies and mean values taken.
