# Supplementary material for: Quantifying the relationship between food sharing practices and socio-ecological variables in small-scale societies: A cross-cultural multi-methodological approach
Source: PLoS One. 2019 May 29;14(5):e0216302. doi: 10.1371/journal.pone.0216302 (PMC6541262; doi:10.1371/journal.pone.0216302)
Supplement: S2 Appendix — (DOCX) [file pone.0216302.s006.docx]

**S2 Appendix – Multiple comparison corrections**

Alpha correction according to Bonferroni: 0.000170068

Alpha correction according to Šidàk: 0.0001744518

|  |  | **Corrected *p*-values** | | | | |
| --- | --- | --- | --- | --- | --- | --- |
|  | **ordered *p*-values** | **Holm correction** | **Hochberg correction** | **Hommel correction** | **Benjamini & Hochberg correction** | **Benjamini & Yekutieli correction** |
| 1 | 0.00003 | 0.01073 | 0.01073 | 0.01073 | 0.01073 | 0.06782 |
| 2 | 0.00205 | 0.63773 | 0.63568 | 0.62748 | 0.21326 | 1 |
| 3 | 0.00205 | 0.63773 | 0.63568 | 0.62748 | 0.21326 | 1 |
| 4 | 0.00303 | 0.93719 | 0.93719 | 0.92506 | 0.23657 | 1 |
| 5 | 0.00380 | 1 | 1 | 1 | 0.23729 | 1 |
| 6 | 0.00519 | 1 | 1 | 1 | 0.23763 | 1 |
| 7 | 0.00533 | 1 | 1 | 1 | 0.23763 | 1 |
| 8 | 0.00725 | 1 | 1 | 1 | 0.27913 | 1 |
| 9 | 0.00805 | 1 | 1 | 1 | 0.27913 | 1 |
| 10 | 0.01716 | 1 | 1 | 1 | 0.53529 | 1 |
| 11 | 0.02077 | 1 | 1 | 1 | 0.58903 | 1 |
| 12 | 0.02577 | 1 | 1 | 1 | 0.62264 | 1 |
| 13 | 0.02618 | 1 | 1 | 1 | 0.62264 | 1 |
| 14 | 0.02794 | 1 | 1 | 1 | 0,62264 | 1 |
| 15 | 0.03764 | 1 | 1 | 1 | 0.71314 | 1 |
| 16 | 0.04067 | 1 | 1 | 1 | 0.71314 | 1 |
| 17 | 0.04346 | 1 | 1 | 1 | 0.71314 | 1 |
| 18 | 0.04531 | 1 | 1 | 1 | 0.71314 | 1 |
| 19 | 0.04762 | 1 | 1 | 1 | 0.71314 | 1 |
| 20 | 0.04769 | 1 | 1 | 1 | 0.71314 | 1 |
| 21 | 0.04800 | 1 | 1 | 1 | 0.71314 | 1 |
| 22 | 0.05032 | 1 | 1 | 1 | 0.71369 | 1 |
| 23 | 0.05833 | 1 | 1 | 1 | 0.75826 | 1 |
| 24 | 0.05833 | 1 | 1 | 1 | 0.75826 | 1 |
| 25 | 0.07205 | 1 | 1 | 1 | 0.85289 | 1 |
| 26 | 0.07587 | 1 | 1 | 1 | 0.85289 | 1 |
| 27 | 0.07682 | 1 | 1 | 1 | 0.85289 | 1 |
| 28 | 0.09173 | 1 | 1 | 1 | 0.85289 | 1 |
| 29 | 0.09615 | 1 | 1 | 1 | 0.85289 | 1 |
| 30 | 0.09815 | 1 | 1 | 1 | 0.85289 | 1 |
| 31 | 0.10008 | 1 | 1 | 1 | 0.85289 | 1 |
| 32 | 0.10109 | 1 | 1 | 1 | 0.85289 | 1 |
| 33 | 0.11420 | 1 | 1 | 1 | 0.85289 | 1 |
| 34 | 0.11592 | 1 | 1 | 1 | 0.85289 | 1 |
| 35 | 0.11715 | 1 | 1 | 1 | 0.85289 | 1 |
| 36 | 0.11784 | 1 | 1 | 1 | 0.85289 | 1 |
| 37 | 0.11784 | 1 | 1 | 1 | 0.85289 | 1 |
| 38 | 0.11784 | 1 | 1 | 1 | 0.85289 | 1 |
| 39 | 0.12295 | 1 | 1 | 1 | 0.85289 | 1 |
| 40 | 0.12295 | 1 | 1 | 1 | 0.85289 | 1 |
| 41 | 0.12588 | 1 | 1 | 1 | 0.85289 | 1 |
| 42 | 0.12642 | 1 | 1 | 1 | 0.85289 | 1 |
| 43 | 0.13493 | 1 | 1 | 1 | 0.85289 | 1 |
| 44 | 0.13493 | 1 | 1 | 1 | 0.85289 | 1 |
| 45 | 0.14081 | 1 | 1 | 1 | 0.85289 | 1 |
| 46 | 0.14081 | 1 | 1 | 1 | 0.85289 | 1 |
| 47 | 0.14081 | 1 | 1 | 1 | 0.85289 | 1 |
| 48 | 0.14165 | 1 | 1 | 1 | 0.85289 | 1 |
| 49 | 0.14438 | 1 | 1 | 1 | 0.85289 | 1 |
| 50 | 0.14438 | 1 | 1 | 1 | 0.85289 | 1 |
| 51 | 0.14478 | 1 | 1 | 1 | 0.85289 | 1 |
| 52 | 0.14504 | 1 | 1 | 1 | 0.85289 | 1 |
| 53 | 0.14640 | 1 | 1 | 1 | 0.85289 | 1 |
| 54 | 0.14762 | 1 | 1 | 1 | 0.85289 | 1 |
| 55 | 0.16236 | 1 | 1 | 1 | 0.89544 | 1 |
| 56 | 0.16359 | 1 | 1 | 1 | 0.89544 | 1 |
| 57 | 0.16359 | 1 | 1 | 1 | 0.89544 | 1 |
| 58 | 0.16655 | 1 | 1 | 1 | 0.89593 | 1 |
| 59 | 0.17222 | 1 | 1 | 1 | 0.91073 | 1 |
| 60 | 0.17689 | 1 | 1 | 1 | 0.91075 | 1 |
| 61 | 0.17806 | 1 | 1 | 1 | 0.91075 | 1 |
| 62 | 0.18514 | 1 | 1 | 1 | 0.92905 | 1 |
| 63 | 0.18760 | 1 | 1 | 1 | 0.92905 | 1 |
| 64 | 0.19522 | 1 | 1 | 1 | 0.92954 | 1 |
| 65 | 0.19584 | 1 | 1 | 1 | 0.92954 | 1 |
| 66 | 0.19987 | 1 | 1 | 1 | 0.92954 | 1 |
| 67 | 0.20957 | 1 | 1 | 1 | 0.92954 | 1 |
| 68 | 0.21841 | 1 | 1 | 1 | 0.92954 | 1 |
| 69 | 0.22183 | 1 | 1 | 1 | 0.92954 | 1 |
| 70 | 0.22693 | 1 | 1 | 1 | 0.92954 | 1 |
| 71 | 0.22693 | 1 | 1 | 1 | 0.92954 | 1 |
| 72 | 0.22693 | 1 | 1 | 1 | 0.92954 | 1 |
| 73 | 0.23485 | 1 | 1 | 1 | 0.92954 | 1 |
| 74 | 0.23649 | 1 | 1 | 1 | 0.92954 | 1 |
| 75 | 0.24880 | 1 | 1 | 1 | 0.92954 | 1 |
| 76 | 0.24880 | 1 | 1 | 1 | 0.92954 | 1 |
| 77 | 0.25130 | 1 | 1 | 1 | 0.92954 | 1 |
| 78 | 0.26000 | 1 | 1 | 1 | 0.92954 | 1 |
| 79 | 0.26214 | 1 | 1 | 1 | 0.92954 | 1 |
| 80 | 0.26220 | 1 | 1 | 1 | 0.92954 | 1 |
| 81 | 0.26220 | 1 | 1 | 1 | 0.92954 | 1 |
| 82 | 0.26220 | 1 | 1 | 1 | 0.92954 | 1 |
| 83 | 0.26242 | 1 | 1 | 1 | 0.92954 | 1 |
| 84 | 0.26242 | 1 | 1 | 1 | 0.92954 | 1 |
| 85 | 0.26659 | 1 | 1 | 1 | 0.92954 | 1 |
| 86 | 0.26669 | 1 | 1 | 1 | 0.92954 | 1 |
| 87 | 0.26815 | 1 | 1 | 1 | 0.92954 | 1 |
| 88 | 0.26934 | 1 | 1 | 1 | 0.92954 | 1 |
| 89 | 0.27135 | 1 | 1 | 1 | 0.92954 | 1 |
| 90 | 0.28149 | 1 | 1 | 1 | 0.92954 | 1 |
| 91 | 0.28884 | 1 | 1 | 1 | 0.92954 | 1 |
| 92 | 0.29734 | 1 | 1 | 1 | 0.92954 | 1 |
| 93 | 0.29840 | 1 | 1 | 1 | 0.92954 | 1 |
| 94 | 0.30021 | 1 | 1 | 1 | 0.92954 | 1 |
| 95 | 0.30067 | 1 | 1 | 1 | 0.92954 | 1 |
| 96 | 0.30503 | 1 | 1 | 1 | 0.92954 | 1 |
| 97 | 0.30654 | 1 | 1 | 1 | 0.92954 | 1 |
| 98 | 0.31005 | 1 | 1 | 1 | 0.92954 | 1 |
| 99 | 0.31015 | 1 | 1 | 1 | 0.92954 | 1 |
| 100 | 0.31426 | 1 | 1 | 1 | 0.92954 | 1 |
| 101 | 0.31806 | 1 | 1 | 1 | 0.92954 | 1 |
| 102 | 0.31928 | 1 | 1 | 1 | 0.92954 | 1 |
| 103 | 0.32052 | 1 | 1 | 1 | 0.92954 | 1 |
| 104 | 0.32373 | 1 | 1 | 1 | 0.92954 | 1 |
| 105 | 0.32808 | 1 | 1 | 1 | 0.92954 | 1 |
| 106 | 0.33028 | 1 | 1 | 1 | 0.92954 | 1 |
| 107 | 0.33206 | 1 | 1 | 1 | 0.92954 | 1 |
| 108 | 0.33383 | 1 | 1 | 1 | 0.92954 | 1 |
| 109 | 0.33640 | 1 | 1 | 1 | 0.92954 | 1 |
| 110 | 0.34012 | 1 | 1 | 1 | 0.92954 | 1 |
| 111 | 0.34204 | 1 | 1 | 1 | 0.92954 | 1 |
| 112 | 0.34436 | 1 | 1 | 1 | 0.92954 | 1 |
| 113 | 0.34628 | 1 | 1 | 1 | 0.92954 | 1 |
| 114 | 0.34890 | 1 | 1 | 1 | 0.92954 | 1 |
| 115 | 0.35519 | 1 | 1 | 1 | 0.92954 | 1 |
| 116 | 0.35748 | 1 | 1 | 1 | 0.92954 | 1 |
| 117 | 0.36503 | 1 | 1 | 1 | 0.92954 | 1 |
| 118 | 0.36503 | 1 | 1 | 1 | 0.92954 | 1 |
| 119 | 0.37056 | 1 | 1 | 1 | 0.92954 | 1 |
| 120 | 0.37663 | 1 | 1 | 1 | 0.92954 | 1 |
| 121 | 0.38097 | 1 | 1 | 1 | 0.92954 | 1 |
| 122 | 0.38370 | 1 | 1 | 1 | 0.92954 | 1 |
| 123 | 0.38398 | 1 | 1 | 1 | 0.92954 | 1 |
| 124 | 0.38498 | 1 | 1 | 1 | 0.92954 | 1 |
| 125 | 0.38636 | 1 | 1 | 1 | 0.92954 | 1 |
| 126 | 0.38741 | 1 | 1 | 1 | 0.92954 | 1 |
| 127 | 0.39507 | 1 | 1 | 1 | 0.92954 | 1 |
| 128 | 0.39594 | 1 | 1 | 1 | 0.92954 | 1 |
| 129 | 0.40077 | 1 | 1 | 1 | 0.92954 | 1 |
| 130 | 0.40651 | 1 | 1 | 1 | 0.92954 | 1 |
| 131 | 0.40686 | 1 | 1 | 1 | 0.92954 | 1 |
| 132 | 0.40686 | 1 | 1 | 1 | 0.92954 | 1 |
| 133 | 0.40686 | 1 | 1 | 1 | 0.92954 | 1 |
| 134 | 0.40779 | 1 | 1 | 1 | 0.92954 | 1 |
| 135 | 0.42044 | 1 | 1 | 1 | 0.92954 | 1 |
| 136 | 0.43282 | 1 | 1 | 1 | 0.92954 | 1 |
| 137 | 0.43368 | 1 | 1 | 1 | 0.92954 | 1 |
| 138 | 0.43418 | 1 | 1 | 1 | 0.92954 | 1 |
| 139 | 0.44082 | 1 | 1 | 1 | 0.92954 | 1 |
| 140 | 0.44284 | 1 | 1 | 1 | 0.92954 | 1 |
| 141 | 0.44471 | 1 | 1 | 1 | 0.92954 | 1 |
| 142 | 0.44558 | 1 | 1 | 1 | 0.92954 | 1 |
| 143 | 0.44558 | 1 | 1 | 1 | 0.92954 | 1 |
| 144 | 0.44558 | 1 | 1 | 1 | 0.92954 | 1 |
| 145 | 0.44558 | 1 | 1 | 1 | 0.92954 | 1 |
| 146 | 0.44610 | 1 | 1 | 1 | 0.92954 | 1 |
| 147 | 0.44747 | 1 | 1 | 1 | 0.92954 | 1 |
| 148 | 0.44747 | 1 | 1 | 1 | 0.92954 | 1 |
| 149 | 0.44747 | 1 | 1 | 1 | 0.92954 | 1 |
| 150 | 0.44790 | 1 | 1 | 1 | 0.92954 | 1 |
| 151 | 0.46364 | 1 | 1 | 1 | 0.92954 | 1 |
| 152 | 0.46364 | 1 | 1 | 1 | 0.92954 | 1 |
| 153 | 0.46808 | 1 | 1 | 1 | 0.92954 | 1 |
| 154 | 0.46842 | 1 | 1 | 1 | 0.92954 | 1 |
| 155 | 0.47032 | 1 | 1 | 1 | 0.92954 | 1 |
| 156 | 0.47073 | 1 | 1 | 1 | 0.92954 | 1 |
| 157 | 0.47073 | 1 | 1 | 1 | 0.92954 | 1 |
| 158 | 0.47073 | 1 | 1 | 1 | 0.92954 | 1 |
| 159 | 0.47501 | 1 | 1 | 1 | 0.93209 | 1 |
| 160 | 0.48217 | 1 | 1 | 1 | 0.94023 | 1 |
| 161 | 0.49021 | 1 | 1 | 1 | 0.94050 | 1 |
| 162 | 0.49290 | 1 | 1 | 1 | 0.94050 | 1 |
| 163 | 0.49290 | 1 | 1 | 1 | 0.94050 | 1 |
| 164 | 0.49673 | 1 | 1 | 1 | 0.94050 | 1 |
| 165 | 0.49738 | 1 | 1 | 1 | 0.94050 | 1 |
| 166 | 0.50439 | 1 | 1 | 1 | 0.94801 | 1 |
| 167 | 0.50783 | 1 | 1 | 1 | 0.94877 | 1 |
| 168 | 0.52472 | 1 | 1 | 1 | 0.96111 | 1 |
| 169 | 0.52517 | 1 | 1 | 1 | 0.96111 | 1 |
| 170 | 0.52517 | 1 | 1 | 1 | 0.96111 | 1 |
| 171 | 0.53487 | 1 | 1 | 1 | 0.96111 | 1 |
| 172 | 0.53807 | 1 | 1 | 1 | 0.96111 | 1 |
| 173 | 0.53807 | 1 | 1 | 1 | 0.96111 | 1 |
| 174 | 0.54914 | 1 | 1 | 1 | 0.96111 | 1 |
| 175 | 0.55004 | 1 | 1 | 1 | 0.96111 | 1 |
| 176 | 0.55304 | 1 | 1 | 1 | 0.96111 | 1 |
| 177 | 0.55559 | 1 | 1 | 1 | 0.96111 | 1 |
| 178 | 0.55559 | 1 | 1 | 1 | 0.96111 | 1 |
| 179 | 0.55744 | 1 | 1 | 1 | 0.96111 | 1 |
| 180 | 0.56319 | 1 | 1 | 1 | 0.96111 | 1 |
| 181 | 0.56533 | 1 | 1 | 1 | 0.96111 | 1 |
| 182 | 0.56591 | 1 | 1 | 1 | 0.96111 | 1 |
| 183 | 0.56973 | 1 | 1 | 1 | 0.96111 | 1 |
| 184 | 0.56989 | 1 | 1 | 1 | 0.96111 | 1 |
| 185 | 0.56989 | 1 | 1 | 1 | 0.96111 | 1 |
| 186 | 0.57933 | 1 | 1 | 1 | 0.96580 | 1 |
| 187 | 0.58241 | 1 | 1 | 1 | 0.96580 | 1 |
| 188 | 0.58571 | 1 | 1 | 1 | 0.96580 | 1 |
| 189 | 0.59270 | 1 | 1 | 1 | 0.96580 | 1 |
| 190 | 0.59357 | 1 | 1 | 1 | 0.96580 | 1 |
| 191 | 0.59459 | 1 | 1 | 1 | 0.96580 | 1 |
| 192 | 0.59863 | 1 | 1 | 1 | 0.96580 | 1 |
| 193 | 0.60053 | 1 | 1 | 1 | 0.96580 | 1 |
| 194 | 0.60053 | 1 | 1 | 1 | 0.96580 | 1 |
| 195 | 0.61886 | 1 | 1 | 1 | 0.97788 | 1 |
| 196 | 0.62730 | 1 | 1 | 1 | 0.97788 | 1 |
| 197 | 0.62774 | 1 | 1 | 1 | 0.97788 | 1 |
| 198 | 0.62976 | 1 | 1 | 1 | 0.97788 | 1 |
| 199 | 0.62980 | 1 | 1 | 1 | 0.97788 | 1 |
| 200 | 0.63344 | 1 | 1 | 1 | 0.97788 | 1 |
| 201 | 0.63528 | 1 | 1 | 1 | 0.97788 | 1 |
| 202 | 0.63549 | 1 | 1 | 1 | 0.97788 | 1 |
| 203 | 0.63625 | 1 | 1 | 1 | 0.97788 | 1 |
| 204 | 0.64021 | 1 | 1 | 1 | 0.97914 | 1 |
| 205 | 0.64806 | 1 | 1 | 1 | 0.98233 | 1 |
| 206 | 0.64859 | 1 | 1 | 1 | 0.98233 | 1 |
| 207 | 0.65208 | 1 | 1 | 1 | 0.98285 | 1 |
| 208 | 0.67265 | 1 | 1 | 1 | 0.99276 | 1 |
| 209 | 0.67364 | 1 | 1 | 1 | 0.99276 | 1 |
| 210 | 0.67578 | 1 | 1 | 1 | 0.99276 | 1 |
| 211 | 0.67965 | 1 | 1 | 1 | 0.99276 | 1 |
| 212 | 0.67965 | 1 | 1 | 1 | 0.99276 | 1 |
| 213 | 0.68532 | 1 | 1 | 1 | 0.99276 | 1 |
| 214 | 0.68677 | 1 | 1 | 1 | 0.99276 | 1 |
| 215 | 0.70058 | 1 | 1 | 1 | 0.99276 | 1 |
| 216 | 0.70434 | 1 | 1 | 1 | 0.99276 | 1 |
| 217 | 0.71818 | 1 | 1 | 1 | 0.99276 | 1 |
| 218 | 0.72442 | 1 | 1 | 1 | 0.99276 | 1 |
| 219 | 0.72542 | 1 | 1 | 1 | 0.99276 | 1 |
| 220 | 0.72563 | 1 | 1 | 1 | 0.99276 | 1 |
| 221 | 0.73086 | 1 | 1 | 1 | 0.99276 | 1 |
| 222 | 0.73086 | 1 | 1 | 1 | 0.99276 | 1 |
| 223 | 0.73423 | 1 | 1 | 1 | 0.99276 | 1 |
| 224 | 0.74299 | 1 | 1 | 1 | 0.99276 | 1 |
| 225 | 0.74375 | 1 | 1 | 1 | 0.99276 | 1 |
| 226 | 0.74375 | 1 | 1 | 1 | 0.99276 | 1 |
| 227 | 0.75207 | 1 | 1 | 1 | 0.99276 | 1 |
| 228 | 0.75682 | 1 | 1 | 1 | 0.99276 | 1 |
| 229 | 0.75818 | 1 | 1 | 1 | 0.99276 | 1 |
| 230 | 0.75925 | 1 | 1 | 1 | 0.99276 | 1 |
| 231 | 0.76160 | 1 | 1 | 1 | 0.99276 | 1 |
| 232 | 0.76395 | 1 | 1 | 1 | 0.99276 | 1 |
| 233 | 0.76395 | 1 | 1 | 1 | 0.99276 | 1 |
| 234 | 0.76697 | 1 | 1 | 1 | 0.99276 | 1 |
| 235 | 0.76730 | 1 | 1 | 1 | 0.99276 | 1 |
| 236 | 0.77078 | 1 | 1 | 1 | 0.99276 | 1 |
| 237 | 0.77430 | 1 | 1 | 1 | 0.99276 | 1 |
| 238 | 0.77430 | 1 | 1 | 1 | 0.99276 | 1 |
| 239 | 0.77430 | 1 | 1 | 1 | 0.99276 | 1 |
| 240 | 0.78310 | 1 | 1 | 1 | 0.99276 | 1 |
| 241 | 0.78326 | 1 | 1 | 1 | 0.99276 | 1 |
| 242 | 0.78326 | 1 | 1 | 1 | 0.99276 | 1 |
| 243 | 0.78326 | 1 | 1 | 1 | 0.99276 | 1 |
| 244 | 0.78527 | 1 | 1 | 1 | 0.99276 | 1 |
| 245 | 0.78701 | 1 | 1 | 1 | 0.99276 | 1 |
| 246 | 0.79262 | 1 | 1 | 1 | 0.99276 | 1 |
| 247 | 0.79376 | 1 | 1 | 1 | 0.99276 | 1 |
| 248 | 0.79600 | 1 | 1 | 1 | 0.99276 | 1 |
| 249 | 0.80527 | 1 | 1 | 1 | 0.99276 | 1 |
| 250 | 0.80691 | 1 | 1 | 1 | 0.99276 | 1 |
| 251 | 0.81540 | 1 | 1 | 1 | 0.99276 | 1 |
| 252 | 0.81677 | 1 | 1 | 1 | 0.99276 | 1 |
| 253 | 0.81800 | 1 | 1 | 1 | 0.99276 | 1 |
| 254 | 0.82008 | 1 | 1 | 1 | 0.99276 | 1 |
| 255 | 0.82034 | 1 | 1 | 1 | 0.99276 | 1 |
| 256 | 0.82120 | 1 | 1 | 1 | 0.99276 | 1 |
| 257 | 0.82120 | 1 | 1 | 1 | 0.99276 | 1 |
| 258 | 0.82120 | 1 | 1 | 1 | 0.99276 | 1 |
| 259 | 0.82667 | 1 | 1 | 1 | 0.99276 | 1 |
| 260 | 0.83746 | 1 | 1 | 1 | 0.99276 | 1 |
| 261 | 0.84201 | 1 | 1 | 1 | 0.99276 | 1 |
| 262 | 0.84391 | 1 | 1 | 1 | 0.99276 | 1 |
| 263 | 0.84721 | 1 | 1 | 1 | 0.99276 | 1 |
| 264 | 0.84834 | 1 | 1 | 1 | 0.99276 | 1 |
| 265 | 0.84926 | 1 | 1 | 1 | 0.99276 | 1 |
| 266 | 0.85436 | 1 | 1 | 1 | 0.99276 | 1 |
| 267 | 0.85714 | 1 | 1 | 1 | 0.99276 | 1 |
| 268 | 0.85714 | 1 | 1 | 1 | 0.99276 | 1 |
| 269 | 0.86746 | 1 | 1 | 1 | 0.99276 | 1 |
| 270 | 0.86755 | 1 | 1 | 1 | 0.99276 | 1 |
| 271 | 0.86755 | 1 | 1 | 1 | 0.99276 | 1 |
| 272 | 0.86755 | 1 | 1 | 1 | 0.99276 | 1 |
| 273 | 0.87036 | 1 | 1 | 1 | 0.99276 | 1 |
| 274 | 0.87185 | 1 | 1 | 1 | 0.99276 | 1 |
| 275 | 0.87982 | 1 | 1 | 1 | 0.99568 | 1 |
| 276 | 0.88079 | 1 | 1 | 1 | 0.99568 | 1 |
| 277 | 0.89895 | 1 | 1 | 1 | 1 | 1 |
| 278 | 0.90203 | 1 | 1 | 1 | 1 | 1 |
| 279 | 0.90215 | 1 | 1 | 1 | 1 | 1 |
| 280 | 0.90226 | 1 | 1 | 1 | 1 | 1 |
| 281 | 0.91429 | 1 | 1 | 1 | 1 | 1 |
| 282 | 0.91893 | 1 | 1 | 1 | 1 | 1 |
| 283 | 0.92444 | 1 | 1 | 1 | 1 | 1 |
| 284 | 0.93068 | 1 | 1 | 1 | 1 | 1 |
| 285 | 0.93281 | 1 | 1 | 1 | 1 | 1 |
| 286 | 0.93370 | 1 | 1 | 1 | 1 | 1 |
| 287 | 0.93610 | 1 | 1 | 1 | 1 | 1 |
| 288 | 0.94131 | 1 | 1 | 1 | 1 | 1 |
| 289 | 0.94521 | 1 | 1 | 1 | 1 | 1 |
| 290 | 0.94740 | 1 | 1 | 1 | 1 | 1 |
| 291 | 0.94792 | 1 | 1 | 1 | 1 | 1 |
| 292 | 0.95349 | 1 | 1 | 1 | 1 | 1 |
| 293 | 0.95436 | 1 | 1 | 1 | 1 | 1 |
| 294 | 0.95686 | 1 | 1 | 1 | 1 | 1 |
| 295 | 0.95852 | 1 | 1 | 1 | 1 | 1 |
| 296 | 0.96343 | 1 | 1 | 1 | 1 | 1 |
| 297 | 0.96427 | 1 | 1 | 1 | 1 | 1 |
| 298 | 0.96705 | 1 | 1 | 1 | 1 | 1 |
| 299 | 0.96705 | 1 | 1 | 1 | 1 | 1 |
| 300 | 0.96818 | 1 | 1 | 1 | 1 | 1 |
| 301 | 0.97693 | 1 | 1 | 1 | 1 | 1 |
| 302 | 0.97765 | 1 | 1 | 1 | 1 | 1 |
| 303 | 0.98519 | 1 | 1 | 1 | 1 | 1 |
| 304 | 1 | 1 | 1 | 1 | 1 | 1 |
| 305 | 1 | 1 | 1 | 1 | 1 | 1 |
| 306 | 1 | 1 | 1 | 1 | 1 | 1 |
| 307 | 1 | 1 | 1 | 1 | 1 | 1 |
| 308 | 1 | 1 | 1 | 1 | 1 | 1 |
| 309 | 1 | 1 | 1 | 1 | 1 | 1 |
| 310 | 1 | 1 | 1 | 1 | 1 | 1 |
| 311 | 1 | 1 | 1 | 1 | 1 | 1 |
| 312 | 1 | 1 | 1 | 1 | 1 | 1 |

Table B. Corrected *p-*values for the 312 *p-*values obtained with the multiple independent two-sample statistical tests in S2 Table.

The corrected *p-*values appear in Table B arranged in ascending order. Considering a significance level of 0.05, we can just reject the null hypothesis of equality of means between groups for the test where the *p-*value equals 0.00003, i.e., percentage of dependence on animal husbandry and status distribution.
