# Supplementary material for: Quantifying the relationship between food sharing practices and socio-ecological variables in small-scale societies: A cross-cultural multi-methodological approach
Source: PLoS One. 2019 May 29;14(5):e0216302. doi: 10.1371/journal.pone.0216302 (PMC6541262; doi:10.1371/journal.pone.0216302)
Supplement: S3 Appendix — (DOCX) [file pone.0216302.s007.docx]

**S3 Appendix – HHG corrected *p*-values in ascending order**

1. Correction of the first *p*-value: pval.hhg.sc, obtained for the sum of Pearson chi-squared statistics from the 2x2 contingency tables considered

|  | **ordered HHG pval.hhg.sc** | **Bonferroni correction** | **Holm correction** | **Hochberg correction** |
| --- | --- | --- | --- | --- |
| Hamming distance in sharing practices | 0.0010 | 0.0230 | 0.0230 | 0.0230 |
| Temperature constancy difference | 0.0809 | 1 | 1 | 0.9650 |
| Agriculture difference | 0.0839 | 1 | 1 | 0.9650 |
| Temperature contingency difference | 0.0889 | 1 | 1 | 0.9650 |
| Elevation difference | 0.1079 | 1 | 1 | 0.9650 |
| Annual net primary production variance difference | 0.2038 | 1 | 1 | 0.9650 |
| Precipitation contingency difference | 0.2058 | 1 | 1 | 0.9650 |
| Annual precipitation variance difference | 0.2138 | 1 | 1 | 0.9650 |
| Slope difference | 0.2138 | 1 | 1 | 0.9650 |
| Annual mean temperature difference | 0.2288 | 1 | 1 | 0.9650 |
| Annual mean precipitation difference | 0.2567 | 1 | 1 | 0.9650 |
| Gathering difference | 0.3207 | 1 | 1 | 0.9650 |
| Annual temperature variance difference | 0.3616 | 1 | 1 | 0.9650 |
| Hunting difference | 0.4016 | 1 | 1 | 0.9650 |
| Monthly mean net primary production difference | 0.4176 | 1 | 1 | 0.9650 |
| Animal husbandry difference | 0.4246 | 1 | 1 | 0.9650 |
| Net primary production constancy difference | 0.4406 | 1 | 1 | 0.9650 |
| Net primary production contingency difference | 0.4745 | 1 | 1 | 0.9650 |
| Distance to coast difference | 0.5065 | 1 | 1 | 0.9650 |
| Precipitation constancy difference | 0.5534 | 1 | 1 | 0.9650 |
| Population size difference | 0.6723 | 1 | 1 | 0.9650 |
| Fishing difference | 0.8412 | 1 | 1 | 0.9650 |
| Geographic distances | 0.9650 | 1 | 1 | 0.9650 |

Table C. HHG corrected *p-*values for the 23 *p-*values obtained for the sum of Pearson chi-squared statistics from the 2x2 contingency tables considered.

The original *p-*values (p.val.hhg.sc) and its corresponding corrections appear arranged in ascending order. Considering a significance level of 0.05, no significant relationship is found except for the Hamming distance with itself, which has no implications at all.

1. Correction of the second *p*-value: pval.hhg.sl, obtained for the sum of the likelihood ratio ("G statistic") values from the 2x2 tables considered.

|  | **ordered HHG pval.hhg.sl** | **Bonferroni correction** | **Holm correction** | **Hochberg correction** |
| --- | --- | --- | --- | --- |
| Hamming distance in sharing practices | 0.0010 | 0.0230 | 0.0230 | 0.0230 |
| Temperature constancy difference | 0.0639 | 1 | 1 | 0.9530 |
| Agriculture difference | 0.0659 | 1 | 1 | 0.9530 |
| Temperature contingency difference | 0.0789 | 1 | 1 | 0.9530 |
| Elevation difference | 0.0959 | 1 | 1 | 0.9530 |
| Precipitation contingency difference | 0.2018 | 1 | 1 | 0.9530 |
| Annual net primary production variance difference | 0.2068 | 1 | 1 | 0.9530 |
| Annual precipitation variance difference | 0.2248 | 1 | 1 | 0.9530 |
| Slope difference | 0.2398 | 1 | 1 | 0.9530 |
| Annual mean temperature difference | 0.2707 | 1 | 1 | 0.9530 |
| Annual mean precipitation difference | 0.2757 | 1 | 1 | 0.9530 |
| Gathering difference | 0.3417 | 1 | 1 | 0.9530 |
| Annual temperature variance difference | 0.3816 | 1 | 1 | 0.9530 |
| Hunting difference | 0.4046 | 1 | 1 | 0.9530 |
| Monthly mean net primary production difference | 0.4156 | 1 | 1 | 0.9530 |
| Net primary production constancy difference | 0.4466 | 1 | 1 | 0.9530 |
| Animal husbandry difference | 0.4496 | 1 | 1 | 0.9530 |
| Net primary production contingency difference | 0.4785 | 1 | 1 | 0.9530 |
| Distance to coast difference | 0.5085 | 1 | 1 | 0.9530 |
| Precipitation constancy difference | 0.5724 | 1 | 1 | 0.9530 |
| Population size difference | 0.6723 | 1 | 1 | 0.9530 |
| Fishing difference | 0.8641 | 1 | 1 | 0.9530 |
| Geographic distances | 0.9530 | 1 | 1 | 0.9530 |

Table D. HHG corrected *p*-values for the 23 *p*-values obtained for the sum of the likelihood ratio (“G statistic) values from the 2x2 contingency tables considered.

The original *p-*values (p.val.hhg.sl) and its corresponding corrections appear arranged in ascending order. Considering a significance level of 0.05, no significant relationship is found except for the Hamming distance with itself, which has no implications at all.

1. Correction of the third *p*-value: pval.hhg.mc, obtained as the maximum Pearson chi-squared statistic from any of the 2x2 tables.

|  | **ordered HHG pval.hhg.mc** | **Bonferroni correction** | **Holm correction** | **Hochberg correction** |
| --- | --- | --- | --- | --- |
| Hamming distance in sharing practices | 0.0010 | 0.0230 | 0.0230 | 0.0230 |
| Temperature contingency difference | 0.0729 | 1 | 1 | 0.9600 |
| Elevation difference | 0.1259 | 1 | 1 | 0.9600 |
| Hunting difference | 0.1269 | 1 | 1 | 0.9600 |
| Annual precipitation variance difference | 0.1838 | 1 | 1 | 0.9600 |
| Agriculture difference | 0.1958 | 1 | 1 | 0.9600 |
| Annual temperature variance difference | 0.2178 | 1 | 1 | 0.9600 |
| Population size difference | 0.2498 | 1 | 1 | 0.9600 |
| Annual net primary production variance difference | 0.3057 | 1 | 1 | 0.9600 |
| Slope difference | 0.4396 | 1 | 1 | 0.9600 |
| Precipitation constancy difference | 0.5145 | 1 | 1 | 0.9600 |
| Net primary production contingency difference | 0.5564 | 1 | 1 | 0.9600 |
| Animal husbandry difference | 0.5674 | 1 | 1 | 0.9600 |
| Gathering difference | 0.5754 | 1 | 1 | 0.9600 |
| Annual mean temperature difference | 0.5954 | 1 | 1 | 0.9600 |
| Net primary production constancy difference | 0.6603 | 1 | 1 | 0.9600 |
| Precipitation contingency difference | 0.6793 | 1 | 1 | 0.9600 |
| Annual mean precipitation difference | 0.7073 | 1 | 1 | 0.9600 |
| Fishing difference | 0.7942 | 1 | 1 | 0.9600 |
| Temperature constancy difference | 0.8012 | 1 | 1 | 0.9600 |
| Distance to coast difference | 0.8022 | 1 | 1 | 0.9600 |
| Geographic distances | 0.8042 | 1 | 1 | 0.9600 |
| Monthly mean net primary production difference | 0.9600 | 1 | 1 | 0.9600 |

Table E. HHG corrected *p-*value*s* for the 23 *p*-values obtained as the maximum Pearson chi-squared statistic from any of the 2x2 contingency tables considered.

The original *p-*values (p.val.hhg.mc) and its corresponding corrections appear arranged in ascending order. Considering a significance level of 0.05, no significant relationship is found except for the Hamming distance with itself, which has no implications at all.

1. Correction of the fourth *p*-value: pval.hhg.ml, obtained as the maximum *G* statistic from any of the 2x2 tables.

|  | **ordered HHG pval.hhg.ml** | **Bonferroni correction** | **Holm correction** | **Hochberg correction** |
| --- | --- | --- | --- | --- |
| Hamming distance in sharing practices | 0.0010 | 0.0230 | 0.0230 | 0.0230 |
| Agriculture difference | 0.0310 | 0.7123 | 0.6813 | 0.6813 |
| Net primary production contingency difference | 0.0719 | 1 | 1 | 0.9700 |
| Temperature contingency difference | 0.1528 | 1 | 1 | 0.9700 |
| Hunting difference | 0.1918 | 1 | 1 | 0.9700 |
| Annual precipitation variance difference | 0.3357 | 1 | 1 | 0.9700 |
| Net primary production constancy difference | 0.3357 | 1 | 1 | 0.9700 |
| Annual temperature variance difference | 0.4316 | 1 | 1 | 0.9700 |
| Annual net primary production variance difference | 0.4535 | 1 | 1 | 0.9700 |
| Population size difference | 0.4745 | 1 | 1 | 0.9700 |
| Precipitation contingency difference | 0.4915 | 1 | 1 | 0.9700 |
| Elevation difference | 0.4955 | 1 | 1 | 0.9700 |
| Precipitation constancy difference | 0.5135 | 1 | 1 | 0.9700 |
| Animal husbandry difference | 0.5784 | 1 | 1 | 0.9700 |
| Temperature constancy difference | 0.6204 | 1 | 1 | 0.9700 |
| Slope difference | 0.6783 | 1 | 1 | 0.9700 |
| Distance to coast difference | 0.7932 | 1 | 1 | 0.9700 |
| Annual mean temperature difference | 0.8142 | 1 | 1 | 0.9700 |
| Gathering difference | 0.8252 | 1 | 1 | 0.9700 |
| Annual mean precipitation difference | 0.8751 | 1 | 1 | 0.9700 |
| Fishing difference | 0.9091 | 1 | 1 | 0.9700 |
| Monthly mean net primary production difference | 0.9191 | 1 | 1 | 0.9700 |
| Geographic distances | 0.9700 | 1 | 1 | 0.9700 |

Table F. **HHG corrected *p*-values for the 23 *p*-values obtained as the maximum G statistic from any of the 2x2 contingency tables considered.**

The original *p-*values (p.val.hhg.ml) and its corresponding corrections appear arranged in ascending order. Considering a significance level of 0.05, no significant relationship is found except for the Hamming distance with itself, which has no implications at all.

It is interesting to note that each of the four calculations of *p*-values gives different values for each variable. Therefore, when arranging them in ascending order, the order in which the environmental/economic variables appear is different in each case (first column from tables C to F).
